# Light Dynamics of the Retinal‐Disease‐Relevant G90D Bovine Rhodopsin Mutant†

**DOI:** 10.1002/anie.202003671

**Published:** 2020-08-13

**Authors:** Nina Kubatova, Jiafei Mao, Carl Elias Eckert, Krishna Saxena, Santosh L. Gande, Josef Wachtveitl, Clemens Glaubitz, Harald Schwalbe

**Affiliations:** ^1^ Center for Biomolecular Magnetic Resonance Goethe University Frankfurt Max-von-Laue-Strasse 7 60438 Frankfurt Germany; ^2^ Institute for Organic Chemistry and Chemical Biology Goethe University Frankfurt Max-von-Laue-Strasse 7 60438 Frankfurt Germany; ^3^ Institute of Biophysical Chemistry Goethe University Frankfurt Max-von-Laue-Strasse 9 60438 Frankfurt Germany; ^4^ Institute of Physical and Theoretical Chemistry Goethe University Frankfurt Max-von-Laue-Strasse 7 60438 Frankfurt Germany

**Keywords:** G-protein-coupled receptors, NMR spectroscopy, retinal, rhodopsin, UV/Vis spectroscopy

## Abstract

The *RHO* gene encodes the G‐protein‐coupled receptor (GPCR) rhodopsin. Numerous mutations associated with impaired visual cycle have been reported; the G90D mutation leads to a constitutively active mutant form of rhodopsin that causes CSNB disease. We report on the structural investigation of the retinal configuration and conformation in the binding pocket in the dark and light‐activated state by solution and MAS‐NMR spectroscopy. We found two long‐lived dark states for the G90D mutant with the 11‐*cis* retinal bound as Schiff base in both populations. The second minor population in the dark state is attributed to a slight shift in conformation of the covalently bound 11‐*cis* retinal caused by the mutation‐induced distortion on the salt bridge formation in the binding pocket. Time‐resolved UV/Vis spectroscopy was used to monitor the functional dynamics of the G90D mutant rhodopsin for all relevant time scales of the photocycle. The G90D mutant retains its conformational heterogeneity during the photocycle.

## Graphical abstract

Rhodopsin is the major dim light receptor in vertebrate eyes. Numerous mutations associated with impaired visual cycles are known. The G90D mutation leads to a constitutively active mutant form of rhodopsin that causes congenital stationary night blindness (CSNB). We investigated the consequences of this mutation on the visual cycle, both in terms of structural aspects and dynamic changes.

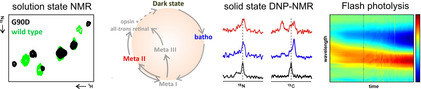

## Introduction

Rhodopsin, an archetypical G‐protein‐coupled receptor (GPCR), belongs to the most studied G‐protein coupled transmembrane receptor family. The retinal chromophore, a derivative of vitamin A, is a key player in the photocycle of rhodopsin. It is bound to opsin^1^ as Schiff base with the side chain of K296 and is stabilized by the side chain of the counter ion E113.^2^ Upon illumination, the retinal cofactor undergoes a 11‐*cis* to all‐*trans* isomerization, which induces a conformational change of rhodopsin, resulting in several intermediate states of the photocycle that can be distinguished by UV/Vis spectroscopy.^3^ The light‐activated Meta II state, in which the retinal is bound in the all‐*trans* conformation, initiates the photo‐transduction cascade.^4^ This state is linked to the deprotonation of the Schiff base that results in an absorption maximum shift to 380 nm and is coupled to the disruption of a salt bridge with the side chain of E113 that becomes protonated indicating a proton transfer in the hydrophobic binding pocket.^5^ This initial photochemical step leads to the largest conformational changes of rhodopsin in the photocycle accompanied by opening of the G protein binding site followed by the transducin activation.^4^, ^6^

In contrast to bacteriorhodopsin, the photocycle of mammalian rhodopsin involves retinal release and uptake and results in decay into opsin and free all‐*trans* retinal. The decay of rhodopsin to opsin and free retinal can proceed through two alternative pathways with different kinetics. Relaxation via the Meta II state is characterized by a deprotonated all‐*trans* retinal and a protonated counter ion E113. With a duration of five minutes, this process is five times faster than the relaxation via the Meta III state (25 minutes).^7^ In contrast to Meta II, the Meta III state is characterized by a protonated retinal in all‐*trans*‐15‐*syn* conformation with an absorption maximum of 465 nm. Since this pathway is significantly slower than the Meta II decay, the Meta III state has been proposed to act as a storage conformation of inactive rhodopsin. Approximately 40 % of the thermal relaxation takes place via this slow kinetic pathway populating the Meta III state. In contrast to this light‐activated Meta II state, which is directly involved in signaling by interaction with visual G protein, the Meta III state is inactive. The light‐induced helical rearrangements mostly affect the secondary structure elements located in the cytoplasmic region, which are crucial for the activation of the G protein. Furthermore, while the structure of the rhodopsin Meta II state is well studied (pdb: 3pxo),^4^ the Meta III state remains poorly understood and is often even not mentioned in the publications.

Changes at any step of the photocycle can impair the visual cycle and therefore lead to numerous visual disorders.^8^ A hotspot for mutations of the wild type sequence is amino acid G90. Depending on the nature of the introduced amino acid, mutations of G90 can either lead to the most common human‐inherited retinal dystrophy night blindness disease called *retinitis pigmentosa* (RP)^8^ or congenital stationary night blindness (CSNB)^9^, ^10^ (G90V and G90D, respectively). CSNB is a non‐progressive inherited retinal disorder, which was found to be genetically and clinically heterogeneous. First symptoms of this disease are the reduction of dim and night vision, problems with the adaptation to darkness and in some cases loss of the general visual acuity. CSNB exhibits an overlapping phenotype with visual diseases such as RP, progressive rod‐cone dystrophy and acquired night blindness (vitamin A deficiency) but in contrast to these diseases, CSNB is non‐progressive.^11^ Currently, there are no preventive measures for this disease, similar to RP. Gene therapy^12^ and photoreceptor transplantations^13]^ are possible future cures, which are under development.

All CSNB relevant mutations are located in the retinal binding pocket and lead to constitutively active rhodopsin mutants.^14^, ^15^, ^16^, ^17^ Crystal structures of the constitutively active G90D mutant are only available for the ligand‐free opsin conformation and the light‐activated state (pdb: 4bez).^18^ So far, no structural data of the G90D mutant in the dark state has been reported and a comprehensive characterization of the retinal binding pocket could not yet be achieved. In their paper, Standfuss and co‐workers argue that structural heterogeneity due to the presence of opsin and rhodopsin prevents crystallization. Further, they propose that the ground state of the G90D mutant is destabilized due to the E113‐K296 Schiff base disruption that would lead to increased rate of retinal thermal isomerization. Although the light active conformation of the G90D mutant was shown to be stabilized and structurally very similar to the wild type Meta II conformation, the conducted crystallographic refinement of the binding pocket indicated a mixture of non‐covalently bound retinal *cis* isomers.

Here, we present a detailed structural characterization employing liquid‐ and solid‐state NMR together with time‐resolved optical spectroscopy to contribute to our understanding of the disease‐induced basal activity of G90D mutant rhodopsin.^15^ In accordance with X‐ray data performed by Standfuss and co‐workers, the G90D mutant with the thermal stabilizing disulfide bond in the extracellular side (N2C/D282C) was investigated.^18^, ^19^, ^20^

## Results and Discussion

### Influence of the Retinal Binding on the Folding Propensity of the Protein

We conducted our experiments by investigating three different rhodopsin constructs: (i) the wild type (WT) construct, (ii) the stabilized wild type with a N2C/D282C double mutation (WT_S‐S_) and the stabilized CSNB‐related G90D mutant (G90D_S‐S_). The double mutation N2C/D282C introduces an additional disulfide bond on the extracellular side between the N‐terminus and the loop E3, which increases the thermal stability of the protein without significantly affecting its activity and structure.^19^, ^20^

However, these mutations impede the retinal reconstitution efficiency and require optimization in the HEK293 expressed rhodopsin purification strategy. In contrast to the wild type purification, where opsin was reconstituted with 11‐*cis* retinal for four hours before the extraction from the cellular membrane,^7^ the opsin of the G90D mutant was incorporated with excess of 11‐*cis* retinal overnight only after its solubilization in DDM detergent and binding to antibodies. Successful 11‐*cis* retinal incorporation to the G90D opsin was confirmed by the characteristic absorption maximum at 490 nm^15^, ^18^ that corresponds to the dark state rhodopsin (Figure S3).

**Figure 1:**
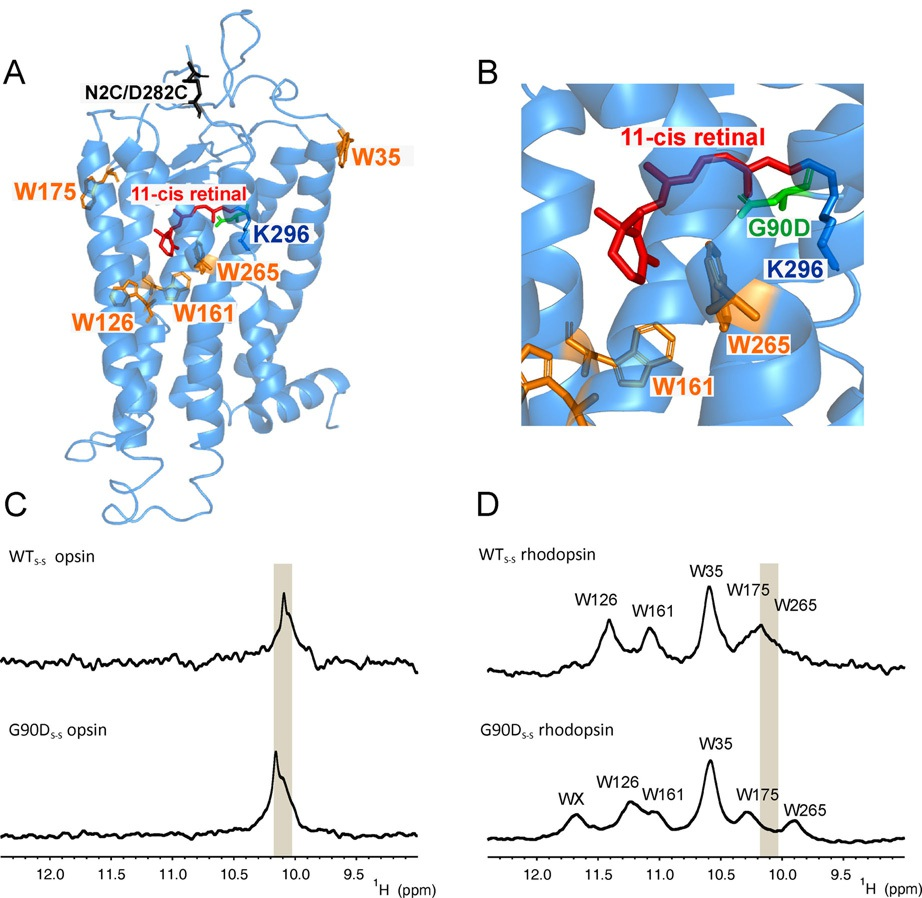
A) Crystal structure of WT_S‐S_ in the dark state (pdb: 2j4y) with modeled G90D mutation. The five tryptophan residues are highlighted as orange sticks, G90D mutation is colored in green, N2C/D282C is highlighted in black and the retinal is shown in red. B) Zoom in the retinal binding pocket of the protein. C) ^1^H 1D NMR spectra of the indole region recorded under dim light conditions of retinal free opsins WT_S‐S_ and G90D_S‐S_ mutant. D) ^1^H 1D NMR spectra of the indole region recorded under dim light conditions of retinal‐bound rhodopsin WT_S‐S_ and G90D_S‐S_ mutant. Resonances of opsin indole signals are highlighted in grey box.

Retinal binding has a crucial effect on the overall structure of the protein, as monitored by chemical shift dispersion of the tryptophan side chain indole resonances by liquid‐state NMR. The retinal‐free opsin conformations of WT_S‐S_ and G90D_S‐S_ show poorly resolved signals at 10.1 ppm of 1D ^1^H NMR spectrum, while the retinal‐bound ground states display well resolved indole signals, indicating significant structural rearrangements and proper folding of the protein (Figure 1).

### Liquid‐State NMR Experiments

**Figure 2:**
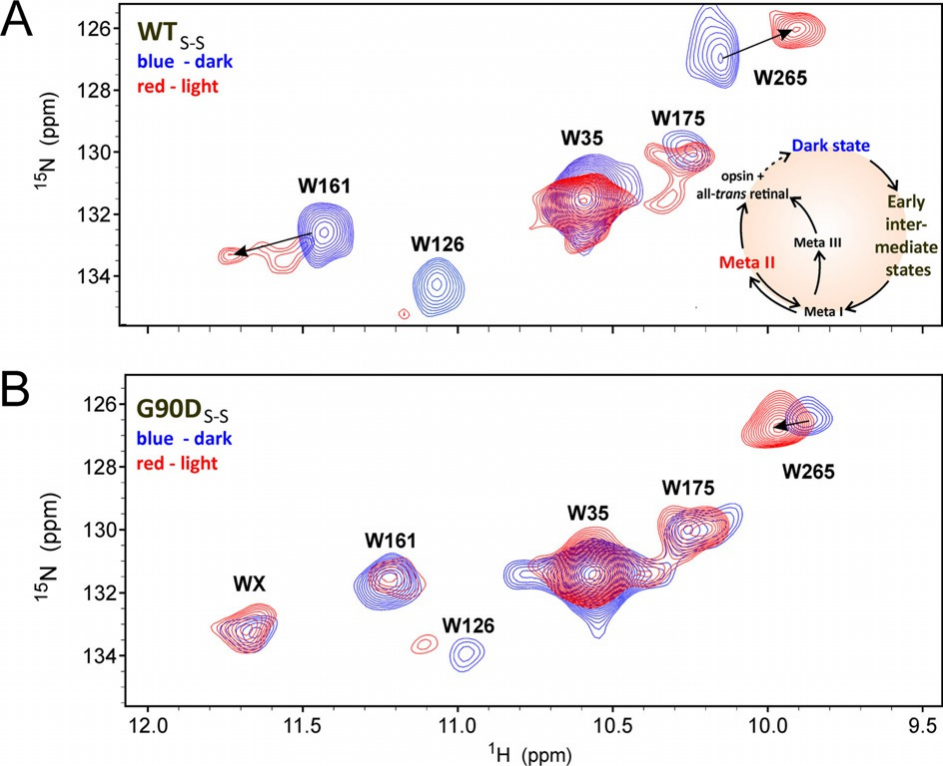
Comparison of the 2D SOFAST‐HMQC ^1^H,^15^N NMR spectra of rhodopsin constructs in the dark and light states. All spectra were recorded at 600 MHz and 298 K. The dark state is colored in blue and the light state is shown in red. Light state experiments started after complete illumination, that was monitored by changes in the absorption maximum at 500 nm and 490 nm for WT_S‐S_ and G90D_S‐S_ respectively, were performed under light exposure. The experiments have been conducted for 8 h. A) Stabilized wild type (WT_S‐S_). B) Stabilized G90D mutant (G90D_S‐S_). Arrows indicate CSPs of W161 and W265. The additional tryptophan signal in the G90D_S‐S_ mutant is labelled WX, tentative assignments are discussed in the main text.

Five tryptophan residues in the rhodopsin sequence were selectively ^15^N isotope labeled using a stably transfected cell line in HEK293 cells and used as reporter signals in liquid‐state NMR experiments. Residues W126^3.41^, W161^4.50^ and W265^6.48^ are highly conserved among GPCRs. They are located in the *trans*‐membrane region and are involved in light‐induced conformational changes.^1^ The tryptophan signals are sensitive to light‐induced conformational changes of the protein, resulting in chemical shift perturbations (CSPs) of the light active conformation.^7^ Monitoring NMR signals in 2D SOFAST‐HMQC ^1^H,^15^N NMR experiments for the different constructs thus provides a direct readout for potential conformational changes. No difference in the indole resonances between WT and WT_S‐S_ could be detected, indicating no effect of the N2C/D282C mutation on the ground state (Figure S4). However, the stabilizing effect of the disulfide bridge was clearly observed on the illuminated rhodopsin conformation that, in contrast to WT, did not aggregate and remained stable (Figure 2 A).

The light‐induced photocycle of the not‐stabilized wild type is completed within 25 minutes, resulting in opsin and free all‐*trans* retinal.^7^ Retinal release from the binding pocket is irreversible and leads to sample aggregation of opsin, accompanied with the decrease and vanishing of all tryptophan signal intensities. Due to the low sample concentration, all 2D spectra of WT_S‐S_ and G90D_S‐S_ were recorded within several hours, which is beyond the photocycle regime. This is in contrast to previously published spectra by Stehle et al., where the high sample concentration allowed us to run experiments for shorter times and signals for the light state could be recorded.^7^

The tryptophan resonances of WT_S‐S_ in the light state are in agreement with the chemical shift assignment of WT in the Meta II state.^7^ Three residues located in the transmembrane region (W265^6.48^, W161^4.50^ and W126^3.41^) are sensitive to the light‐induced conformational changes and show characteristic Meta II CSPs, while two other tryptophan amino acids from the extracellular domain (W35^1.30^ and W175^4.65^) do not undergo any chemical shift perturbations and are resistant to the protein structural rearrangements.

Remarkably, the G90D_S‐S_ mutant shows significant CSPs of the W161^4.50^ and W265^6.48^ signals and, more importantly, an additional signal at 11.65 ppm (labelled WX in Figure 2 B). Taking into account that rhodopsin has five tryptophan residues, the additional sixth tryptophan signal indicates the presence of two long‐lived states of the protein. We cloned and expressed the following triple mutants G90D_S‐S_ (W161F) and G90D_S‐S_ (W265F) to try to assign the additional sixth signal. This W‐F mutation strategy could be successfully applied to assign the 5 tryptophan reporter signals in WT rhodopsin.^21^ In fact, the protein can be cloned and expressed (Figure S1). However, in the G90D_S‐S_ mutant series, the additionally introduced mutation leads to lower affinities of retinal to the triple mutant protein, and as a result, reconstitution and purification of these mutants was not possible.

Interestingly, unlike WT_S‐S_, the G90D_S‐S_ NMR signals of tryptophan residues do not change upon light activation (Figure 2 B). Even W265, which is located in the binding pocket and is highly sensitive to the retinal isomerization, shifts downfield (vs. upfield shift of WT) much less compared to the WT_S‐S_.

The additional WX signal at 11.65 ppm also does not undergo significant CSPs in the dark and light states, showing an almost identical chemical shift compared to the light induced downfield shift of the W161 from WT_S‐S_ in Meta II state. W161^4.50^ is located in the middle of H4, which is not directly involved in the light‐induced conformational changes. But due to the direct connection to H3, which undergoes large structural rearrangements, this residue is co‐affected by the light exposure and is involved indirectly in the Meta II formation. This suggests the Meta II‐origin of the additional sixth signal from the G90D_S‐S_ mutant, which supports the hypothesis of a pre‐active conformation of the G90D_S‐S_ mutant in the dark state.

Based on its location, G90D would be expected to lead to a disruption of the ligand binding pocket, which in turn could result in different long‐lived ground state populations.

### Solid‐State MAS‐NMR Experiments

#### Dark State Solid‐State NMR Experiments

In order to gain experimental evidence for a disrupted binding pocket, DNP‐enhanced (Dynamic Nuclear Polarisation) solid‐state MAS (magic angle spinning) NMR experiments were performed to characterize configuration and conformation of the retinal‐Schiff base complex. The protein was therefore ^15^N lysine isotope labeled and reconstituted with ^13^C_2_ (C14,15) or ^13^C_3_ (C12,13,20)‐retinal (Figure S2). Proteoliposomes were prepared with DOPC (1,2‐dioleoyl‐sn‐glycero‐3‐phosphocholine).

Retinal binding to K296 in opsin takes place via a Schiff base, which causes a characteristic chemical shift change for the ^15^N‐Lys resonance from around 40 to 183 ppm (pSB‐protonated Schiff base, value re‐referenced to liquid ammonium). Upon Schiff base deprotonation, a further shift by 127 ppm is expected.^22^

**Figure 3:**
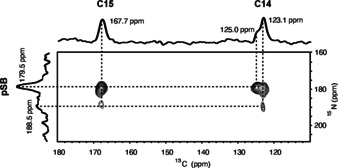
DNP‐enhanced ^15^N‐^13^C TEDOR spectrum of ^13^C_2_‐retinal within the ^15^N‐Lys‐G90D_S‐S_ mutant in the dark state. The 2D spectrum is overlaid in the F2‐dimension with a ^13^C DQF spectrum and in the 1D dimension with a ^15^N‐CP spectrum. The chemical shifts of C14 and C15 could be transferred from Patel et al.^23^ See text for further details.

The 2D ^15^N‐^13^C‐TEDOR (Transferred‐Echo DOuble Resonance) spectrum of ^15^N‐ G90D_S‐S_ with ^13^C_2_‐retinal shows characteristic crosspeaks between a ^15^N pSB resonance at 179.5 ppm and both retinal carbons C14 (125.0 ppm) and C15 (167.7 ppm) providing clear evidence for the Schiff base formation and therefore, for the retinal being covalently bound to K296 (Figure 3). The pSB chemical shift is similar to that observed for the wild type and no resonance for a deprotonated SB species is observed (Figure S8). Downfield to the main pSB signal a shoulder around 188.5 ppm can be detected, which could arise from a lowly populated second conformation. This second conformation shows additional crosspeaks with C14 and C15 (Figure 3). The resonance of the retinal carbon C14 also shows a minor conformation at 125.0 ppm (shoulder) for which, however, no additional crosspeak with the pSB could be detected within the signal‐to‐noise limitations of this experiment.

In order to test whether the observed conformational heterogeneity can also be detected for other retinal carbons, the WT_S‐S_ and the G90D_S‐S_ mutant was reconstituted with ^13^C_3_ (C12,13,20)‐retinal. Here, positions C12, C13 and C20 were chosen as the most light‐sensitive retinal carbons, which are in close proximity to the Schiff base.

**Figure 4:**
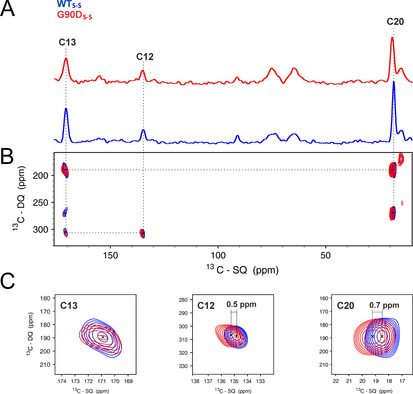
Comparison of DNP‐enhanced spectra of ^13^C_3_‐retinal in G90D_S‐S_ mutant with WT_S‐S_ in the dark state. A) DNP‐enhanced, double quantum‐filtered (DQF) ^13^C spectrum of 12,13,20‐^13^C_3_ retinal in the WT_S‐S_ (blue) and G90D_S‐S_ mutant (red). B) DNP‐enhanced, double quantum‐single quantum (DQ‐SQ) spectra of 12,13,20‐^13^C retinal. C) Zoom in of C12, C13, and C20 retinal signals from DQ‐SQ spectra. Significant CSPs are observed for C12 (changing by 0.5 ppm from 134.8 to 135.3 ppm) and for C20 (changing from 18.5 ppm to 19.2 ppm), while C13 remains at 170.4 ppm.

A comparison between double quantum filtered (DQF) ^13^C‐spectra of WT_S‐S_ and G90D_S‐S_ is shown in Figure 4 A,B. The chemical shift assignment was deduced from the 2D double quantum‐single quantum (DQ‐SQ) correlation experiment (Figure 4 B,C) and is consistent with Patel et al.^23^ However, in contrast to C13, whose chemical shift (170.4 ppm) remains similar for both constructs, C12 and C20 of G90D_S‐S_ show significant downfield CSPs of 0.5 and 0.7 ppm with respect to WT_S‐S_.

#### Light State Solid‐State NMR Experiments

Further characterization of the retinal conformational changes induced by illumination and analysis of the impact of the G90D mutation on the binding pocket geometry was performed in situ in the MAS rotor under cryogenic conditions at 100 K followed by thermal relaxation. Illuminating the sample at 100 K for two hours with blue light allowed trapping of the early photoproduct bathorhodopsin,^24^ while its subsequent warming to room temperature led to the light active Meta II state conformation.^23^

**Figure 5:**
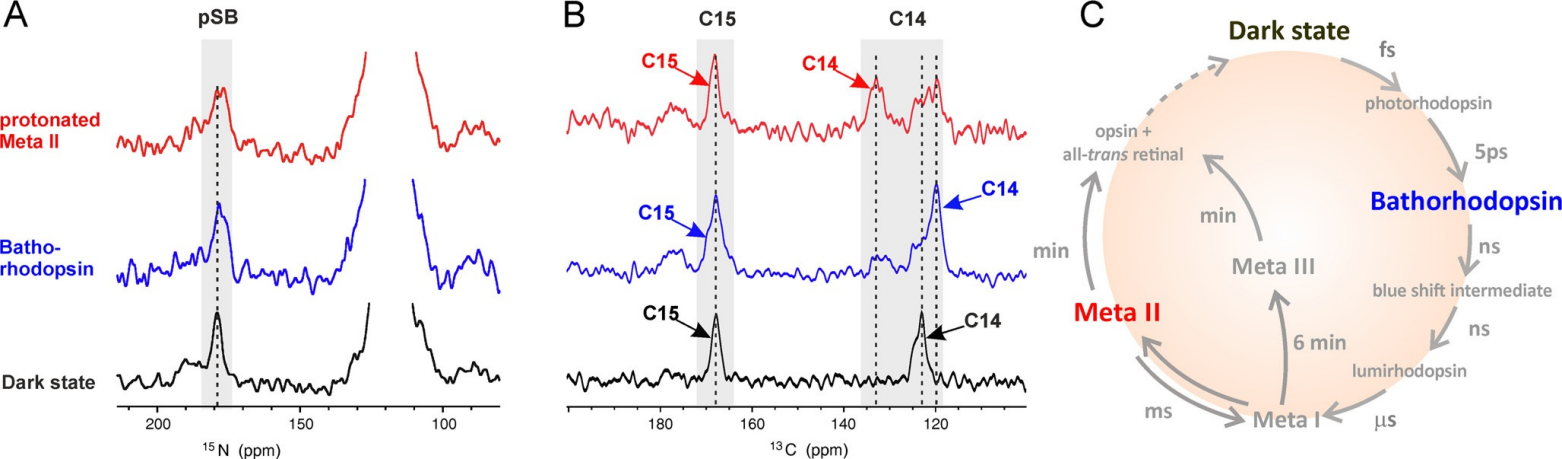
DNP‐Enhanced MAS‐NMR spectra of the G90D_S‐S_ mutant under illumination. Three states were detected: dark state (black), bathorhodopsin (blue), and light active Meta II (red) as discussed in the main text. A) DNP‐enhanced, ^15^N spectrum of ^15^N lysine isotope labeled protein. The signal at 179.5 ppm, which corresponds to the protonated Schiff base, is visible in every state. B) DNP‐enhanced double quantum‐filtered (DQF) ^13^C spectrum of 14,15‐^13^C_2_ retinal. C14 and C15 signal areas are highlighted in gray. For each signal corresponding to the respective state, the same color code was used. C) Photocycle of rhodopsin. Color code for three detected states is in accordance with (A) and (B).

Similar to the dark state, the batho and Meta II states show characteristic protonated Schiff base signals at 179.5 ppm. For both intermediates, the pSB signal profiles are broader than for the dark state, with an additional shoulder shifted 3 ppm upfield (Figure 5 A). However, the low signal‐to‐noise ratio does not allow unambiguous assignment of these shapes to the second conformational population. The signal in the protonated SB region (179.5 ppm) and the absence of any signal in the deprotonated SB area (Figure S8) indicates that the Meta II conformation of the G90D_S‐S_ mutant is protonated and not deprotonated as in WT rhodopsin.

For the analysis of the conformational changes of the retinal under light exposure the retinal carbons C14 and C15 were chosen as reporters. The DQF spectrum of cryo‐trapped G90D_S‐S_ bathorhodopsin reveals a new C14 signal appearing at a chemical shift of 119.8 ppm, which is consistent with the WT batho state.^24^ The detectable minor signal at 123 ppm is assigned to a residual dark state population which remained due to an incomplete illumination process. Carbon C14 in light adapted Meta II state of the G90D_S‐S_ mutant shows conformational heterogeneity with residual signals corresponding to the dark state (123 ppm) and bathorhodopsin (119.8 ppm; Figure 5 B,C).

The Meta II formation is confirmed by a new signal resonating at 132.6 ppm, which is in agreement with downfield shifted wild type Meta II C14 resonance.^23^ A closer examination of C14 DQF spectra shows that even in batho‐rhodopsin a low intensity Meta II signal around 120 ppm is visible. This was not observed for the WT^25^ and might indicate the presence of the light active state in earlier stages of the photocycle, which could possibly result from the pre‐active conformation in the dark state. Furthermore, the aldehyde carbon C15 of non‐covalently bound retinal would resonate around 190 ppm.^26^ The absence of any signal in this range provides clear evidence that only bound retinal is detected in the dark state. The weak spectral feature around 177 ppm occurring in illuminated samples may indicate the presence of an additional minor retinal species.

### Time‐Resolved Optical Spectroscopy

**Figure 6:**
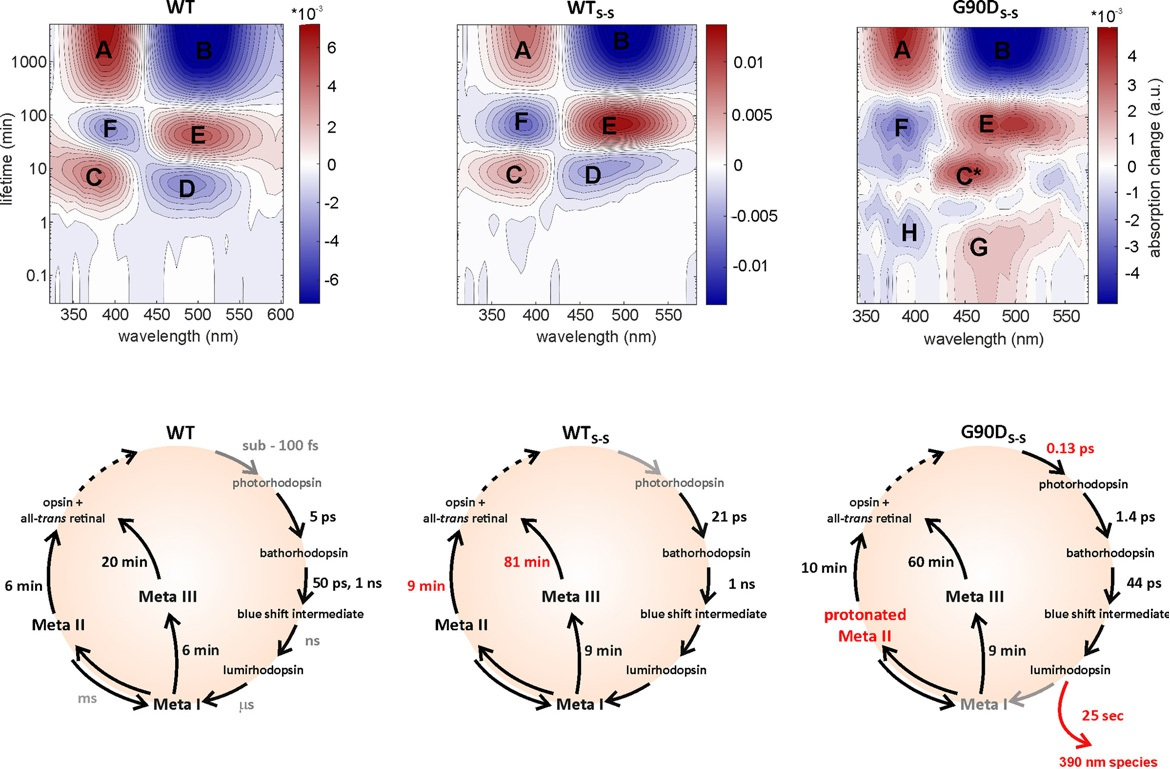
Kinetic analysis of three constructs. Top: Lifetime density maps of the transient absorption data. Positive (red) amplitudes account for decay of absorption, negative (blue) amplitudes account for rise of absorption. Signature A represents free retinal absorption and B stands for the ground‐state bleaching. Both belong to the long‐lived non‐decaying components, which are similar for each sample and mark the end of the photodynamics. Shorter lifetime components C and D describe Meta II decay and Meta III formation, respectively. E is assigned to the longer lifetime Meta III decay and F corresponds to the free retinal formation,^28^ G describes the photo‐intermediate state lumirhodopsin, H is the partial retinal release, and C* is the protonated Meta II decay. Bottom: Schematic representation of the analysis of the lifetimes. Estimated lifetimes reported in the literature but not measured on these constructs are shown in grey.

We further investigated the effect of the G90D mutation on the rhodopsin photocycle by the kinetic experiments. The time‐resolved absorption measurements comprise kinetics from picoseconds up to two hours and provide information on the early retinal isomerization processes up to the evolution of the light active Meta II and Meta III intermediate states. Kinetic experiments detected a four‐fold slower bathorhodopsin state formation of the stabilized wild type. Flash photolysis also reports a stabilizing effect of the N2C/D282C mutation. Thus, the decay of the intermediate states, Meta II and Meta III, is significantly delayed, the covalently bound retinal is stabilized and its hydrolysis is prolonged (Figure 6).

The G90D_S‐S_ mutant shows a strongly altered photocycle compared to WT^7^ and WT_S‐S_, resulting in a unique signature pattern presented in a lifetime density map (LDM) profile.^27^, ^28^ In contrast to the wild type, the photorhodopsin conformation was detectable for the G90D_S‐S_ mutant, indicating the delayed retinal isomerization (Figure S9). The next transition to the bathorhodopsin occurred faster and relaxed more rapidly, resulting in a longer lived batho state. Unlike WT_S‐S_, G90D_S‐S_ mutant shows a weak absorption decay at 0.4 minutes at approximately 490 nm,^29^ which is assigned to the photo‐intermediate state lumirhodopsin (signature G). This decay is accompanied by an absorption increase at 390 nm, which is due to the formation of a blue shifted intermediate (signature H) that appears before the active intermediate states are formed. Furthermore, intermediate Meta III formation and Meta II decay of the G90D_S‐S_ mutant are in the same temporal regime as for the WT_S‐S_, while Meta III decay is 20 minutes faster. Finally, the Meta II state of the G90D_S‐S_ mutant was found in the spectrally slightly blue‐shifted protonated form (signature C*) confirming the results from solid‐state NMR.

Taken together, the N2C/D282C mutations stabilize the light active conformation of rhodopsin delaying the relaxation of its Meta states. The photocycle of the G90D_S‐S_ mutant is unique, showing a protonated Meta II state and an untypical retinal behavior: retinal isomerization is delayed, while the early appearance of a blue shifted component might indicate structural distortion or even transient deprotonation before the active Meta‐intermediates are formed.

## Discussion

### Conformational Heterogeneity

Both from liquid‐state and solid‐state NMR spectroscopy, we find evidence for conformational mixture of two long‐lived states of the CSNB‐related G90D mutant in the dark state comprising a second minor population of the Schiff base and attached retinal. Structural heterogeneity consisting of a mixture of opsin and rhodopsin conformations of the G90D mutant in the dark state was proposed by Singhal et al. as a reason for not obtaining crystals in the dark state. In addition to that we suppose that the heterogeneity is caused by a second minor populated protein conformation with incorporated 11‐*cis* retinal. Retinal conformational heterogeneity was observed only on the C14 carbon and is referred to the slightly different steric position of 11‐*cis* retinal in the binding pocket. Furthermore, we identify the crucial effect of the retinal binding on the overall protein structure. Unlike for the stabilized wild type WT_S‐S_, liquid‐state NMR results of the G90D mutant indicated no significant structural changes of the tryptophan signals between dark and light active conformations, which is consistent with previous results obtained by Fourier‐transform infrared (FTIR),^30^ electron paramagnetic resonance (EPR),^5^ and dynamic single‐molecule force spectroscopy (SMFS)^31^ experiments. Moreover, unlike the wild type, the Meta II conformation of the G90D mutant exists in the protonated Schiff base form fully consistent with the FTIR analysis performed by Zvyaga et al.^30^

### Changes in the Structure and Photocycle of the G90D Mutant

The rhodopsin photocycle is triggered by light‐induced chromophore isomerization. In the dark inactive state, 11‐*cis* retinal is covalently bound to K296 via a protonated Schiff base. An important stabilizing role plays the negatively charged E113 residue, which acts as a counter ion for the protonated Schiff base in the dark state. 11‐*cis* to all‐*trans* isomerization induces a series of intermediate photoproducts, resulting in an active Meta II conformation, which in turn is characterized by deprotonated Schiff base and all‐*trans* covalently attached retinal. The proton transfer from the all‐*trans* Schiff base bound retinal to the negatively charged counter ion E113 is a key process in the activation switch and retinal release. An alternative relaxation pathway of the Meta I state involves Meta III conformation, which is considered as a storage conformation of inactive rhodopsin and comprises protonated retinal in its all‐*trans*‐15‐*syn* conformation. Both intermediates, Meta II and Meta III, subsequently decay to opsin and free all‐*trans* retinal. The constitutively active G90D mutation is located in the retinal binding pocket in close proximity to the residues K296 and E113. The charged D90 side chain interacts with the Schiff base K296, perturbing the salt bridge connection between E113 and K296.^15^ However, the exact effect of the induced mutation on the retinal conformation had remained unclear. The G90D mutation induced distortion in the binding pocket starts in the retinal free opsin conformation. The closer steric position of D90 compared to WT counter ion E113 allows the WT unlike salt bridge formation between the Schiff base and the negatively charged D90 side chain, leading to the stabilization of the G90D opsin state. This reflects on the impeded retinal binding efficiency of the G90D mutant. However, in contrast to the RP associated constitutively active mutations, CSNB related mutants are still able to incorporate retinal, retaining the increased basal activity in the ground state. Mutation of glycine to aspartic acid at position 90 leads to a competing salt bridge formation between the negatively charged E113 and G90, reflecting in the structural heterogeneity of the retinal binding pocket. According to our data, we suggest the Schiff base between 11‐*cis* retinal and K296 to be stabilized by both counter ions, E113 and D90.

Light‐induced proton transfer from positively charged protonated retinal Schiff base to the negatively charged counter ion E113 is a part of the rhodopsin activation switch.^5^ In WT, it results in an increased distance between the Schiff base and the side chain carboxyl group of E113 in the light active Meta II state.^4^ According to the crystal structure of the light adapted Meta II state of G90D mutant,^18^ the distance between K296 and E113 of G90D mutant is similar to the Meta II WT, while D90 is still in close proximate to form the salt bridge and prevent the retinal from its further hydrolysis. Taken together, similar to the WT, upon illumination retinal isomerizes to the all‐*trans* conformation, which is still bound via a Schiff base to the protein. However, the G90D mutation disrupts the hydrogen network, leaving the Meta II state protonated and, therefore, stabilized. In contrast to the wild type, where the retinal isomerization is essential for the corresponding intermediate states and is directly related to the activation of the transduction process, the G90D mutation disrupt this structural cascade withdrawing the antagonistic function of the retinal.

### Origin of the Constitutive Activation

Constitutively active rhodopsin mutations can be classified into two groups. In the first group, changes in the retinal binding pocket and accompanying disturbance of retinal binding and/or retinal release (E113Q,^32^, ^33^ K296,^34^ A292E,^14^ G90D^15^, ^18^ and T94I^16^, ^35^) are proposed. In the second group, the mutated amino acids are located close to the cytoplasmic side and influences the transducin binding site, which is responsible for the G protein activation (M257Y^36^). An increased basal activity is observed for both diseases (RP and CSNB). However, it appears unlikely that this increased activity is the only reason for their distinct phenotypes. Different mutations of the Schiff base K296^34^ and of its counter ion E113Q^32^ lead to a constitutive active state of rhodopsin (RP), while four other constitutive active single point mutations A292E,^14^ G90D,^15^ T94I^16^ and quiet recently discovered A295V mutation^17^ are known to cause CSNB. Furthermore, the nature of the mutated amino acid at a specific position can define the pathology, thus G90V leads to RP while G90D causes CSNB.^9^, ^10^

Three theories were proposed to explain the increased basal activity of the G90D mutant: an active opsin conformation was proposed to be able to activate G protein transducin in the absence of light,^15^, ^37^ an increased thermal isomerization of the retinal in the absence of light^38^ and pre‐active conformation in the dark state. Already previously, the first two models remained ambiguous, leading to conflicts with several reported studies.^30^, ^39^, ^40^

Here, we present several arguments that strongly support the pre‐active conformation of the G90D mutant.

With liquid state NMR experiments, we find a crucial influence of 11‐*cis* retinal binding on the folding properties of the G90D mutant. DNP enhanced solid‐state NMR experiments reported only 11‐*cis* retinal bound to the G90D protein in the dark state, excluding the effect of spontaneous isomerization in the ground state. The second retinal population observed in the dark state does not originate from the light active form and is attributed to the slightly different steric position of the 11‐*cis* retinal. Absence of spontaneous isomerization is supported by the ultrafast absorption spectroscopy that showed the delayed light‐induced retinal isomerization of the early photoproducts of the G90D mutant compared to the WT.

The unambiguous evidence for a pre‐active conformation is provided by liquid‐state NMR experiments. The high similarity between the spectra of G90D mutant, recorded under dark and light conditions, is indicative for the third model of the increased basal activity. Furthermore, structural heterogeneity observed by both, liquid‐ and solid‐state NMR, is consistent with the model proposed by Dizhoor et al.^39^

## Conclusion

Taking together, our results revealed important structural information regarding the CSNB related G90D mutant in the dark and light Meta II state. For the first time the retinal conformation is characterized for three protein states: dark state G90D rhodopsin, G90D bathorhodopsin and G90D Meta II state.

First, we demonstrate retinal being bound to the protein via a Schiff base in all detected states. The conformational heterogeneity of the binding pocket of the G90D mutant in the dark state is originated from the slightly different steric position of the covalently bound 11‐*cis* retinal. Furthermore, structural heterogeneity is also reflected on the global protein conformation, which remains similar for both, dark and light state of the G90D mutant. These data in combination with a unique photocycle of the G90D mutant provide evidence for the pre‐active ground state theory as an explanation of the increased basal activity of the mutant and add an important piece of information for the detailed understanding of the molecular mechanism of night blindness disease.

## Conflict of interest

The authors declare no conflict of interest.

## Supporting information

As a service to our authors and readers, this journal provides supporting information supplied by the authors. Such materials are peer reviewed and may be re‐organized for online delivery, but are not copy‐edited or typeset. Technical support issues arising from supporting information (other than missing files) should be addressed to the authors.

Supplementary

## References

[1] O. P. Ernst , K. P. Hofmann , H. W. Choe , J. H. Park , P. Scheerer , Nature 2008, 454, 183–187. 18563085 10.1038/nature07063

[2] K. Palczewski , T. Kumasaka , T. Hori , C. A. Behnke , H. Motoshima , B. A. Fox , I. Le Trong , D. C. Teller , T. Okada , R. E. Stenkamp , Science 2000, 289, 739–745. 10926528 10.1126/science.289.5480.739

[3] O. P. Ernst , F. J. Bartl , ChemBioChem 2002, 3, 968–974. 12362361 10.1002/1439-7633(20021004)3:10<968::AID-CBIC968>3.0.CO;2-Q

[4] H. W. Choe , Y. J. Kim , J. H. Park , T. Morizumi , E. F. Pai , N. Krauss , K. P. Hofmann , P. Scheerer , O. P. Ernst , Nature 2011, 471, 651–655. 21389988 10.1038/nature09789

[5] J. M. Kim , C. Altenbach , M. Kono , D. D. Oprian , W. L. Hubbell , H. G. Khorana , Proc. Natl. Acad. Sci. USA 2004, 101, 12508–12513. 15306683 10.1073/pnas.0404519101 PMC515088

[6] S. O. Smith , Annu. Rev. Biophys. 2010, 39, 309–328. 20192770 10.1146/annurev-biophys-101209-104901

[7] J. Stehle , R. Silvers , K. Werner , D. Chatterjee , S. Gande , F. Scholz , A. Dutta , J. Wachtveitl , J. Klein-Seetharaman , H. Schwalbe , Angew. Chem. Int. Ed. 2014, 53, 2078–2084; 10.1002/anie.201309581 24505031

[8] S. H. Tsang , T. Sharma , Adv. Exp. Med. Biol. 2018, 1085, 125–130. 30578498 10.1007/978-3-319-95046-4_25

[9] J. Neidhardt , D. Barthelmes , F. Farahmand , J. C. Fleischhauer , W. Berger , Invest. Ophthalmol. Visual Sci. 2006, 47, 1630–1635. 16565402 10.1167/iovs.05-1317

[10] D. Toledo , E. Ramon , M. Aguilà , A. Cordomí , J. J. Pérez , H. F. Mendes , M. E. Cheetham , P. Garriga , J. Biol. Chem. 2011, 286, 39993–40001. 21940625 10.1074/jbc.M110.201517 PMC3220564

[11] C. Zeitz , A. G. Robson , I. Audo , Prog. Retinal Eye Res. 2015, 45, 58–110. 10.1016/j.preteyeres.2014.09.001 25307992

[12] E. Waltz , Nat. Biotechnol. 2018, 36, 6–7. 10.1038/nbt0118-6b 29319694

[13] M. J. Seiler , R. B. Aramant , Prog. Retinal Eye Res. 2012, 31, 661–687. 10.1016/j.preteyeres.2012.06.003 PMC3472113 22771454

[14] T. P. Dryja , E. L. Berson , D. D. Oprian , Nat. Genet. 1993, 4, 290–293. 10.1038/ng0793-280 8358437

[15] V. R. Rao , G. B. Cohen , D. D. Oprian , Nature 1994, 367, 639–642. 8107847 10.1038/367639a0

[16] N. Al-Jandal , G. J. Farrar , A. S. Kiang , M. M. Humphries , N. Bannon , J. B. C. Findlay , P. Humphries , P. F. Kenna , Hum. Mutat. 1999, 13, 75–81. 9888392 10.1002/(SICI)1098-1004(1999)13:1<75::AID-HUMU9>3.0.CO;2-4

[17] C. Zeitz , A. K. Gross , D. Leifert , B. Kloeckener-Gruissem , S. D. McAlear , J. Lemke , J. Neidhardt , W. Berger , Invest. Ophthalmol. Visual Sci. 2008, 49, 4105–4114. 18487375 10.1167/iovs.08-1717

[18] A. Singhal , M. K. Ostermaier , S. A. Vishnivetskiy , V. Panneels , K. T. Homan , J. J. G. Tesmer , D. Veprintsev , X. Deupi , V. V. Gurevich , G. F. X. Schertler , J. Standfuss , EMBO Rep. 2013, 14, 520–526. 23579341 10.1038/embor.2013.44 PMC3674435

[19] G. Xie , A. K. Gross , D. D. Oprian , Biochemistry 2003, 42, 1995–2001. 12590586 10.1021/bi020611z

[20] J. Standfuss , G. Xie , P. C. Edwards , M. Burghammer , D. D. Oprian , G. F. X. Schertler , J. Mol. Biol. 2007, 372, 1179–1188. 17825322 10.1016/j.jmb.2007.03.007 PMC2258155

[21] K. Werner , I. Lehner , H. Kaur , C. Christian , C. Glaubitz , H. Schwalbe , J. Klein-Seetharaman , H. G. Khorana , J. Biomol. NMR 2007, 37, 303–312. 17318366 10.1007/s10858-007-9143-0

[22] S. Ahuja , M. Eilers , A. Hirshfeld , E. C. Y. Yan , M. Ziliox , T. P. Sakmar , M. Sheves , S. O. Smith , J. Am. Chem. Soc. 2009, 131, 15160–15169. 19795853 10.1021/ja9034768 PMC2783296

[23] A. B. Patel , E. Crocker , M. Eilers , A. Hirshfeld , M. Sheves , S. O. Smith , Proc. Natl. Acad. Sci. USA 2004, 101, 10048–10053. 15220479 10.1073/pnas.0402848101 PMC454162

[24] M. Concistrè , A. Gansmüller , N. Mclean , O. G. Johannessen , I. Mari , P. H. M. Bovee-geurts , P. Verdegem , J. Lugtenburg , R. C. D. Brown , W. J. Degrip , J. Am. Chem. Soc. 2008, 130, 10490–10491. 18642911 10.1021/ja803801u

[25] M. Concistrè , A. Gansmüller , N. McLean , O. G. Johannessen , I. M. Montesinos , P. H. M. Bovee-Geurts , R. C. D. Brown , W. J. DeGrip , M. H. Levitt , J. Am. Chem. Soc. 2009, 131, 6133–6140. 19354207 10.1021/ja809878c

[26] A. Albeck , N. Livnah , H. Gottlieb , M. Sheves , J. Am. Chem. Soc. 1992, 114, 2400–2411.

[27] C. Slavov , H. Hartmann , J. Wachtveitl , Anal. Chem. 2015, 87, 2328–2336. 25590674 10.1021/ac504348h

[28] D. Chatterjee , C. E. Eckert , C. Slavov , K. Saxena , B. Fürtig , C. R. Sanders , V. V. Gurevich , J. Wachtveitl , H. Schwalbe , Angew. Chem. Int. Ed. 2015, 54, 13555–13560; 10.1002/anie.201505798 PMC4685475 26383645

[29] C. E. Eckert, Doctoral Thesis, Goethe-University Frankfurt, **2017**.

[30] T. A. Zvyaga , K. Fahmy , F. Siebert , T. P. Sakmar , Biochemistry 1996, 35, 7536–7545. 8652533 10.1021/bi960391n

[31] S. Kawamura , A. T. Colozo , L. Ge , D. J. Müller , P. S. H. Park , J. Biol. Chem. 2012, 287, 21826–21835. 22549882 10.1074/jbc.M112.340182 PMC3381145

[32] P. R. Robinson , G. B. Cohen , E. A. Zhukovsky , D. D. Oprian , Neuron 1992, 9, 719–725. 1356370 10.1016/0896-6273(92)90034-b

[33] P. C. Edwards , A. D. Antona , M. Fransen , G. Xie , D. D. Oprian , G. F. X. Schertler , Nature 2011, 471, 656–660. 21389983 10.1038/nature09795 PMC3715716

[34] G. B. Cohen , T. Yang , P. R. Robinson , D. D. Oprian , Biochemistry 1993, 32, 6111–6115. 8099498 10.1021/bi00074a024

[35] A. Singhal , Y. Guo , M. Matkovic , G. F. X. Schertler , X. Deupi , E. C. Y. Yan , J. Standfuss , EMBO Rep. 2016, 17, 1431–1440. 27458239 10.15252/embr.201642671 PMC5048376

[36] X. Deupi , P. Edwards , A. Singhal , B. Nickle , D. Oprian , G. F. X. Schertler , J. Standfuss , Proc. Natl. Acad. Sci. USA 2012, 109, 119–124. 22198838 10.1073/pnas.1114089108 PMC3252945

[37] A. K. Gross , V. R. Rao , D. D. Oprian , Biochemistry 2003, 42, 2009–2015. 12590588 10.1021/bi020613j

[38] P. A. Sieving , J. E. Richards , F. Naarendorp , E. L. Bingham , K. Scorrt , M. Alpern , Proc. Natl. Acad. Sci. USA 1995, 92, 880–884. 7846071 10.1073/pnas.92.3.880 PMC42724

[39] A. M. Dizhoor , M. L. Woodruff , E. V. Olshevskaya , M. C. Cilluffo , M. C. Cornwall , P. A. Sieving , G. L. Fain , J. Neurosci. 2008, 28, 11662–11672. 18987202 10.1523/JNEUROSCI.4006-08.2008 PMC2590870

[40] S. Jin , M. C. Cornwall , D. D. Oprian , Nat. Neurosci. 2003, 6, 731–735. 12778053 10.1038/nn1070

