# Supplementary material for: Light Dynamics of the Retinal‐Disease‐Relevant G90D Bovine Rhodopsin Mutant
Source: Angew Chem Int Ed Engl. 2020 Aug 13;59(36):15656–64. doi: 10.1002/anie.202003671 (PMC7496284; doi:10.1002/anie.202003671)
Supplement: Supplementary file 1 — Supplementary [file ANIE-59-15656-s001.pdf]

Angewandte Chemie
Eine Zeitschrift der Gesellschaft Deutscher Chemiker

Supporting Information

Light Dynamics of the Retinal-Disease-Relevant G90D Bovine Rhodopsin Mutant**

Nina Kubatova[+], Jiafei Mao[+], Carl Elias Eckert, Krishna Saxena, Santosh L. Gande, Josef Wachtveitl, Clemens Glaubitz,* and Harald Schwalbe**

anie_202003671_sm_miscellaneous_information.pdf

Supporting Information

1. Materials and Methods 2
1.1. Construction of expression plasmids for mutant opsin genes 2
1.2. Generation stable cell lines expressing rhodopsin mutants 2
1.3. Expression and purification of rhodopsin constructs 3
1.4. Liquid state NMR experiments 5
1.5. Time-Resolved Absorption Spectroscopy 5
1.6. Solid state NMR experiments and sample preparation 6
2. Results 7
2.1. UV/Vis-absorption spectra 7
2.2. Liquid state NMR experiments 9
2.3. Solid state MAS-NMR experiments 12
3. References 18

1. Materials and Methods

1.1. Construction of expression plasmids for mutant opsin genes

The rhodopsin constructs were expressed using the pACMV-tetO vector (Dr. Philip Reeves [1]). The W161F/G90D/N2C/D282C and the W265F/G90D/N2C/D282C mutations were introduced using the QuikChange II Site-Directed Mutagenesis Kit (Agilent Technologies). Moreover, two other constructs (WT_{S-S} and $G90D_{S-S}$) carrying the stabilizing mutation (N2C/D282C) were kindly provided by Prof. Standfuss (Switzerland) [2].

1.2. Generation stable cell lines expressing rhodopsin mutants

Flp-In™ T-REx™ 293 cells were used for the generation of stable cell lines that enable tetracycline-induced rhodopsin mutant gene expression. Flp-In™ T-REx™ 293 cells were grown in Dulbecco's modified Eagle's medium supplemented with 10% fetal calf serum, 1% penicillin/streptomycin (Invitrogen) and blasticidin (5µg/ ml), under 5% CO_2 at 37 °C. Stable transfections were performed with Lipofectamine 2000 following the protocol recommended by the supplier (Invitrogen). Typically, 4 µg of plasmid DNA were used per 6-cm dish. Transfected cells were grown for 2 weeks in medium containing geneticin (G418 sulfate) with increasing concentrations from 500 to 2000 µg/ml. Drug-resistant single clones were isolated using cloning cylinders and expanded in growth medium containing geneticin (2 mg/ml) [3]. Further, clones were screened for inducible expression of rhodopsin mutant protein, with tetracycline (2 µg/ml) and Na-butyrate (5 mM/ml), by Western blot analysis. Expression profile of different clones as analyzed by immune blotting is shown in the figure below. Healthy positive clones with high expression of protein were selected for further expansion and protein purification.

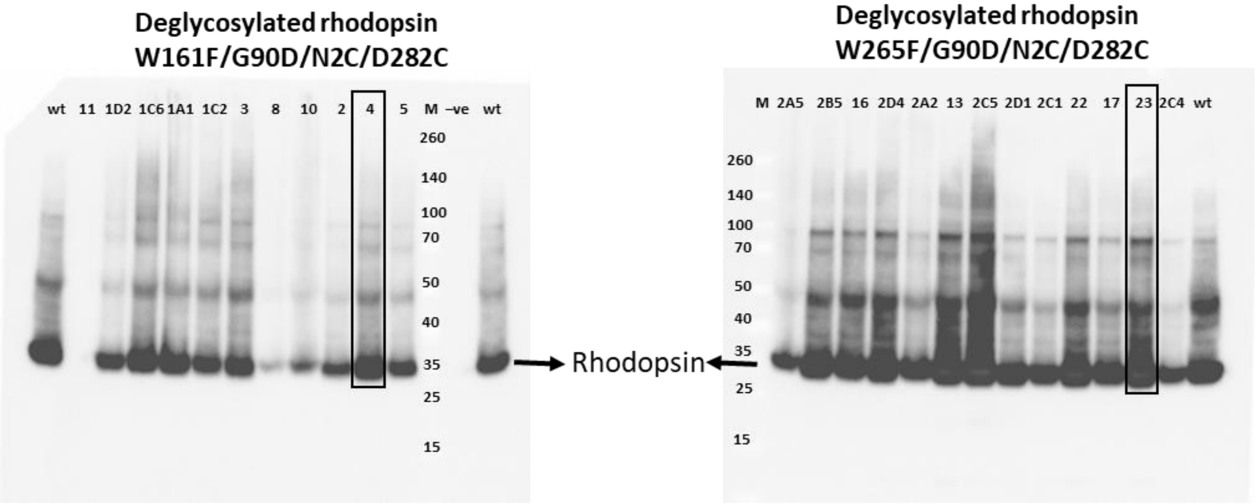

Figure S1. Western blot of rhodopsin constructs. M: Molecular weight marker; wt: wild type rhodopsin Immunoblot: Equal amounts of solubilized and deglycosylated (protein deglycosylation mix II, NEB) total cell extracts of different clones from 6 well plates were loaded on Nu-PAGE™ 4-12% Bis Tris gel (Invitrogen), followed by the protein transfer to PVDF membrane. Immuno detection of rhodopsin mutant proteins was performed using rho 1D4 monoclonal as primary antibody and HRP conjugated goat anti-mouse as secondary antibody (dianova GmbH). Followed by ECL detection using Lumi imager™ F1. Stable clones in the text box were further expanded for protein purification.

1.3. Expression and purification of rhodopsin constructs

Opsin constructs were expressed in tetracycline inducible HEK293 cells. For the large scale expression the cells were grown in an incubator with 5% CO_2 at 37°C in 143 cm^2 culture petri dishes. The induction of the cells was conducted with medium cell culture medium with the desired isotope selective labeling components. Liquid state NMR experiments were performed using α,ε-^{15}N labeled tryptophan, while solid state NMR experiments included ^{15}N labeled lysine. The samples for the photoflash analysis were prepared with natural abundance DMEM medium. Approximately 150 to 200 petri dishes were required to achieve the minimal sufficient concentration of the sample (ca. 0.7 mg) for liquid and solid state NMR experiments. The photoflash experiments were conducted using around 50 to 70 petri dishes (ca. 80 µg). Cell suspension was supplemented with 11-cis retinal to the final concertation of 20 µM. The 11-cis retinal was achieved by the isomerization of the all-trans isomer followed by the HPLC purification according to [4]. A commercially available all-trans retinal was purified for the liquid state NMR and photoflash analysis, while for the solid state NMR experiments ^{13}C selectively labeled all-trans retinal was used. For ^{13}C labeled retinal two labeling scheme were applied, the first one was ^{13}C isotope labeled at

the positions C12, C13 and C20 (12,13,20-$^{13}C_3$), while the second one was ^{13}C labeled at the C14 and C15 (14,15-$^{13}C_2$) (**Figure S2**).

Wild type suspension was supplemented with 11-cis retinal to the final concertation of 20 µM. The cells were mechanically homogenized and incubated for two hours at 4°C before the second portion of 20 µM 11-cis retinal was added and the incubation was repeated for another two hours. The final concentration of 11-cis retinal was 40 µM [5]. Rhodopsin was extracted from the cellular membrane using 1% DDM (n-dodecyl-b-maltose) as a detergent. Subsequent rhodopsin purification was performed by immunoaffinity chromatography using 1D4 monoclonal antibodies. Therefore, the Rho 1D4 monoclonal antibody (*University of British Columbia, Vancouver, Canada*) was coupled to activated Sepharose 4B (*GE Healthcare)* as described [6]. After rhodopsin was bound to the resin beads, the mixture was washed with 20 mM phosphate buffer (pH 6.5) supplemented with 0.05% DDM. The protein was eluted with nine residues long peptide (TETSQVAPA), which was previously synthesized via solid phase synthesis. Elution buffer was added to the protein solution until no more rhodopsin was detected. The monitoring of the fractions was carried out using UV/Vis spectroscopy. The protein-containing fractions were pooled and concentrated to a final volume of approximately 1 ml (ca. 20 µM).

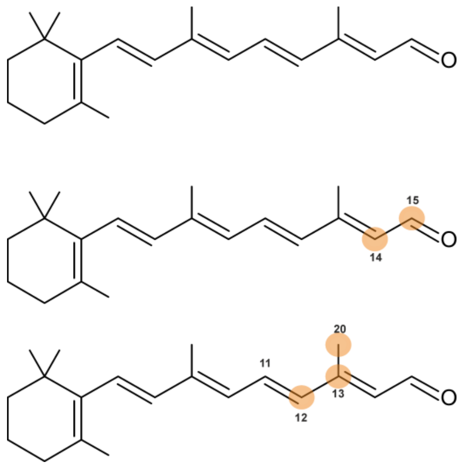

Figure S2. Retinal labeling schemes. 14,15-$^{13}C_2$-retinal ($^{13}C_2$-retinal) and 12,13,20-$^{13}C_3$-retinal ($^{13}C_3$-retinal). Orange circles represent ^{13}C labeling positions.

Rhodopsin constructs containing the stabilizing mutation (N2C/D282C) were purified differently. In contrast to the wild type, these constructs were first extracted from the membrane with 1% DDM and only after binding to antibodies, supplemented with 11-cis retinal [2]. The incorporation with the retinal was performed overnight (*vs*. 4 hours for WT) at 4°C. The washing steps of the rhodopsin remain the

same as those for the wild type, while the elution procedure was slightly changed. The rhodopsin-resin suspension was transferred into a 50 ml falcon tube and supplemented with 12 ml of solution buffer. The mixture was rotated for 45 minutes at 4°C and subsequently collected in one fraction. This step was repeated four times, after which rhodopsin was no longer detected.

1.4. Liquid state NMR experiments

For a liquid state NMR experiments a 20 mM phosphate buffer (pH 6.5) was used. The sample concentration was in the range of 60-80 µM (ca. 0.6-0.8 mg). Liquid state NMR experiments were recorded at 600 MHz and 950 MHz spectrometers at 298 K. 1D x-filter and 2D SOFAST-HMQC experiments were applied to detect α,ε-^{15}N tryptophan labeled indole resonances. Dark state measurements were performed under dim light conditions. NMR kinetics experiments on the $G90D_{S-S}$ mutant were acquired at 800 MHz, 298 K, at different time intervals after illumination with emission wavelength of 514 nm of 300 mW for 0.5 second. All NMR spectra were processed and analyzed using TopSpin version 3.2 (Bruker Biospin).

1.5. Time-Resolved Absorption Spectroscopy

The ***flash photolysis experiments*** have been performed with an optical parametric oscillator (OPO, preciScan, GWU-Lasertechnik) combined with a Nd:YAG laser (Spitlight 600, Innolas Laser GmbH) for the generation of nanosecond pump pulses. The microsecond white light probe pulses were generated by a Xenon flash lamp. For multichannel detection, an intensified charge-coupled device camera (ICCD camera) was used (PI-MAX 3, Princeton Instruments). The samples were photoexcited using a single ns-pulse with a central wavelength of $\lambda_{max} = 500\ nm$. The spectral range of detection was 300-600 nm and the spectral resolution 0.5 nm. The raw data set was wavelength binned resulting in a spectral resolution of ~10 nm. The analysis of the data was performed entirely based on the binned data. The absorption change was detected every 0.5 min within a range from 0 to 120 min. All samples were solubilized in a buffer with DDM at pH 6.5 and RT.

Ultrafast Vis–pump–probe spectroscopy. The setup for time-resolved transient absorption measurements was described previously in detail.[7] It consists of an oscillator/amplifier system (Clark, MXR-CPA-iSeries, 1 kHz, 775 nm 150 fs) which provides the ultrashort laser fundamental pulses. These pulses are then used to drive a home-built two stage NOPA (noncollinear optical parametric amplifier) system [8,9] to produce the excitation pulses used in the experiments. In addition, part of the laser fundamental was focused into a CaF_2-crystal (5 mm) to generate light continuum for the probe pulses. The cross-correlation between the excitation pulses and the probe pulses was ~100 fs. The signal was

detected in reference mode by two spectrometers (600 lines/mm gratings, blazed at 500 nm, and a 64-channel photodiode array). Anisotropic contributions were eliminated by measuring under magic angle conditions (54.7° pump-probe polarization difference). The experiments were carried out in a fused silica cuvette with 1 mm optical path length, which was continuously moved to ensure fresh sample volume for each laser excitation pulse.

Data analysis. The analysis of the experimental data was performed using OPTIMUS (www.optimusfit.org).[10] Lifetime distribution analysis (LDA) was used for the ultrafast transient absorption data as a model independent method of analysis that naturally deals with non-exponential or distributed kinetics. In this analysis, the pre-exponential amplitudes of a set of 100 exponential functions with fixed, equally spaced (on a decimal logarithm scale) lifetimes are determined. The obtained pre-exponential amplitudes at each detection wavelength can be presented in the form of a contour lifetime density map (LDM) [11].

1.6. Solid state NMR experiments and sample preparation

For solid-state NMR experiments, rhodopsin was reconstituted in DOPC (1,2-dioleoyl-sn-glycero-3-phosphocholine) as described before. [12,13] The lipid was added in 100-fold molar excess to the protein and rotated for one hour at 4°C. Subsequently, the proteoliposomes were centrifuged at 52.000 g for one hour at 4°C and incubated with 200 µl AMUPol solution (20 mM, 60% D2O, 30% glycerol-d8, 10% H2O) overnight at 4 °C. The excess of AMUPol solution was removed and the pellet was transferred into a 3.2 mm sapphire MAS NMR rotor under red light. DNP-enhanced NMR experiments were recorded on a Bruker 400 MHz (9.4 T) solid-state NMR spectrometer coupled to a 263 GHz gyrotron microwave source. All spectra were recorded at 110 K and 8 kHz MAS. ^{13}C and ^{15}N chemical shifts were referenced indirectly to DSS and liquid ammonia at room temperature. For all experiments, 100 kHz ^{1}H decoupling using SPINAL-64 was applied during acquisition. ^{1}H-^{15}N-cross polarization (CP) experiments were performed with a contact time of 400 µs and 16k acquisitions. Double quantum filtered 1D ^{13}C spectra were obtained by using the POST-C7 pulse sequence [14] with a double quantum excitation and reconversion time of 0.5 ms and 16k scans. The 2D double-quantum single-quantum (2D-DQSQ) ^{13}C spectra [15] were recorded with 1k scans and 64 t1 increments using a dwell time of 17.86 µs. The DQ-SQ ^{13}C-^{13}C 2D spectra were zero-filled to 8k x 1k data points. A Gaussian window function (lb = -20 Hz and GB = 0.04) was applied for the direct and a Qsine window function (ssb=2) for the indirect dimension. ^{15}N-^{13}C spectra were recorded using a double cross polarization (DCP) experiment. ^{15}N-^{13}C cross polarization was accomplished by matching the rectangular ^{15}N lock pulse (21.3 kHz) with the ramped ^{13}C lock pulse (90%

ramp, max. power 26.7 kHz) during a 4 ms contact time. Spectra were recorded with 4k scans and 24 t1 points (2 kHz F1 window). The proteoliposome samples were illuminated directly within the 3.2 mm thin wall ZrO_2 MAS rotors using a green LED light source a reported in previous works [16,17].

2. Results

2.1. UV/Vis-absorption spectra

UV/Vis-absorption spectroscopy distinguishes between dark and light adapted rhodopsin states. The absorption maximum at 500 nm represents the dark state with 11-cis retinal bound, while an absorption maximum at 380 nm corresponds to the light active Meta II state with all-trans chromophore incorporated to the protein. Remarkably, free retinal absorbs at the same wavelength (380 nm) as Meta II conformation, while retinal free opsin shows only absorption maximum at 280 nm. Due to the mutation caused disruption in the retinal binding pocket, the absorption maximum of the G90D mutant is blue shifted to 490 nm [2]. The concentrations of the wild types and the G90D mutant were calculated according to the light-induced absorption maximum drop at 500 nm and 490 nm, respectively.

Size-exclusion chromatography (SEC) was performed on the G90D mutant to distinguish between the possible origins of the absorption maximum at 380nm observed in the dark state. SEC of the G90D mutant shows two prominent signals with the retention volumes of 15.0 ml and 16.5 ml for the fraction I and II, respectively. First, the absorption profiles were recorded at 280 nm, 380 nm and 480 nm and subsequently observed under dim light condition to avoid illumination. The fractions were collected, concentrated and independently analyzed by absorption spectroscopy. According to the absorption profile, the first fraction (15.0 ml) contains pure rhodopsin and the second fraction (16.5 ml) comprises free retinal. Thus, it was possible to address the absorption maximum at 380 nm to an excess of 11-cis free not bound retinal, remained during the purification steps, and exclude the partial illumination.

Protein purification by size exclusion chromatography (SEC) leads to ca. 50% reduced protein yield. Since the incorporation of 11-cis retinal into the stabilized constructs is impeded under solution NMR sample condition, the excess of unbound retinal is not able to reactivate the photocycle. Therefore, the structure and kinetics of those samples remain unaffected by excess free retinal. This allowed us to omit the SEC purification step and increase the protein amount for liquid state NMR samples.

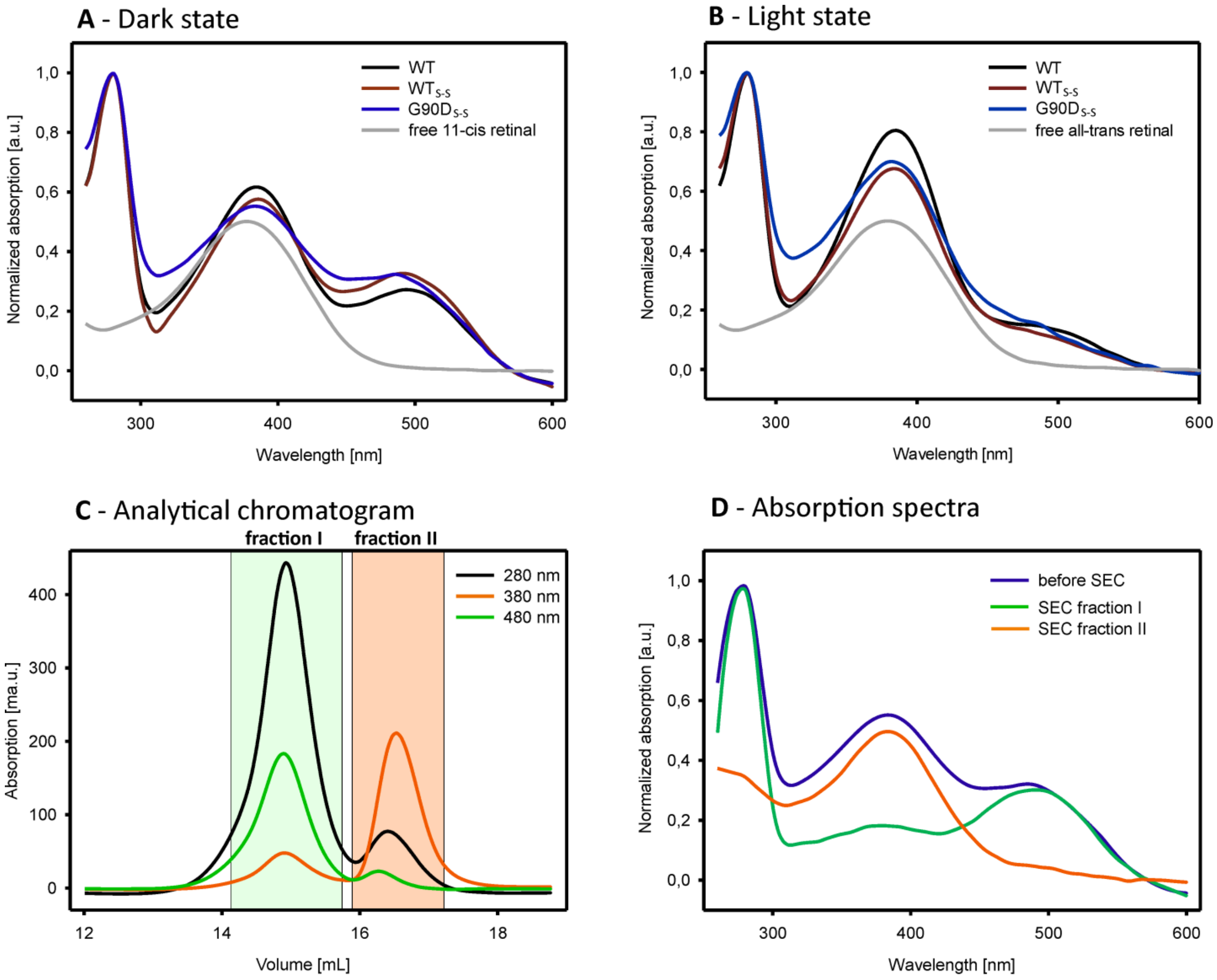

Figure S3. Purification of rhodopsin constructs. A – Absorption spectra of the three constructs and free 11-cis retinal recorded under dim light conditions. The second absorption maximum at 380 nm, which is present in the spectrum of each construct, originates from an excess of 11-cis free not bound retinal that remained during the purification steps. In contrast to WT, incorporation with retinal of the constructs with additional stabilizing double mutation (N2C/N282C) requires optimizes purification strategy (e.g. excess of the retinal). **B** – Absorption spectra of the three constructs and free all-trans retinal after illumination. The absorption spectra are normalized at 280 nm. **C** – Analytical SEC of the G90D mutant performed on Superdex 200 10/300 column. The construct was purified in 1% OG at 4°C under dim light conditions. The collected fractions are highlighted in green (fraction I) and purple (fraction II). **D** – Absorption spectra of the collected fractions and protein before size-exclusion purification. The spectra are normalized at 280 nm.

2.2. Liquid state NMR experiments

The indole resonances observed in 2D SOFAST-HMQC NMR experiment for the WT and $WT_{S\text{-}S}$ show the same signal pattern, allowing to transfer the chemical shift assignment of the wild type performed by Stehle *et al*. [5] to the stabilized construct.

From this experiment it can be inferred that the stabilizing double mutation N2C/D282C does not affect the overall structure of the wild type in the dark state.

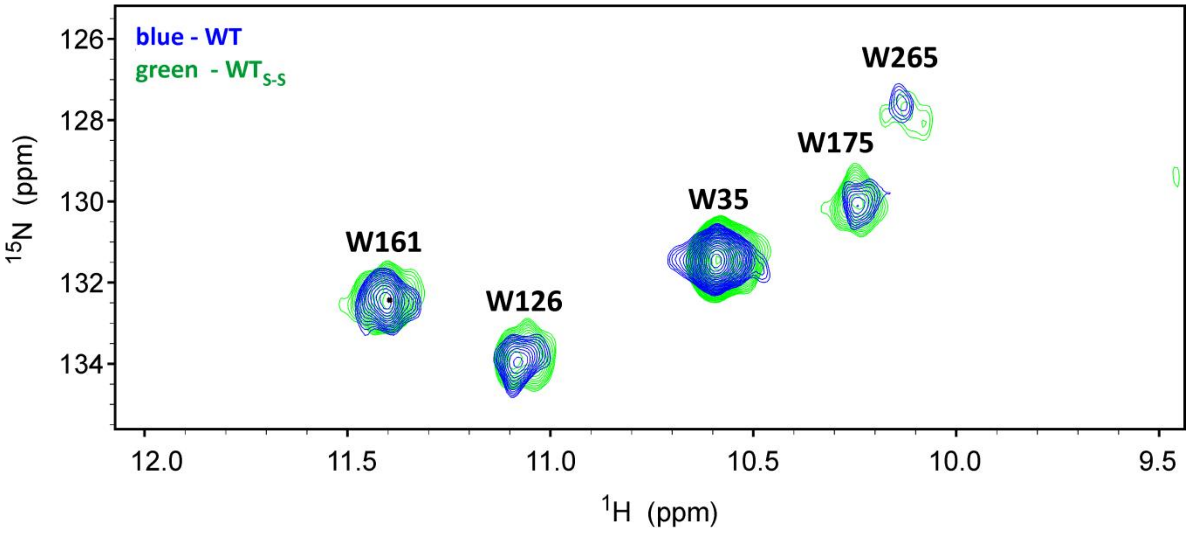

Figure S4. 2D SOFAST-HMQC NMR spectra of rhodopsin constructs in the dark state. All spectra were recorded at 950 MHz, 298 K under light dim conditions. The WT is shown in blue and the $WT_{S\text{-}S}$ in green.

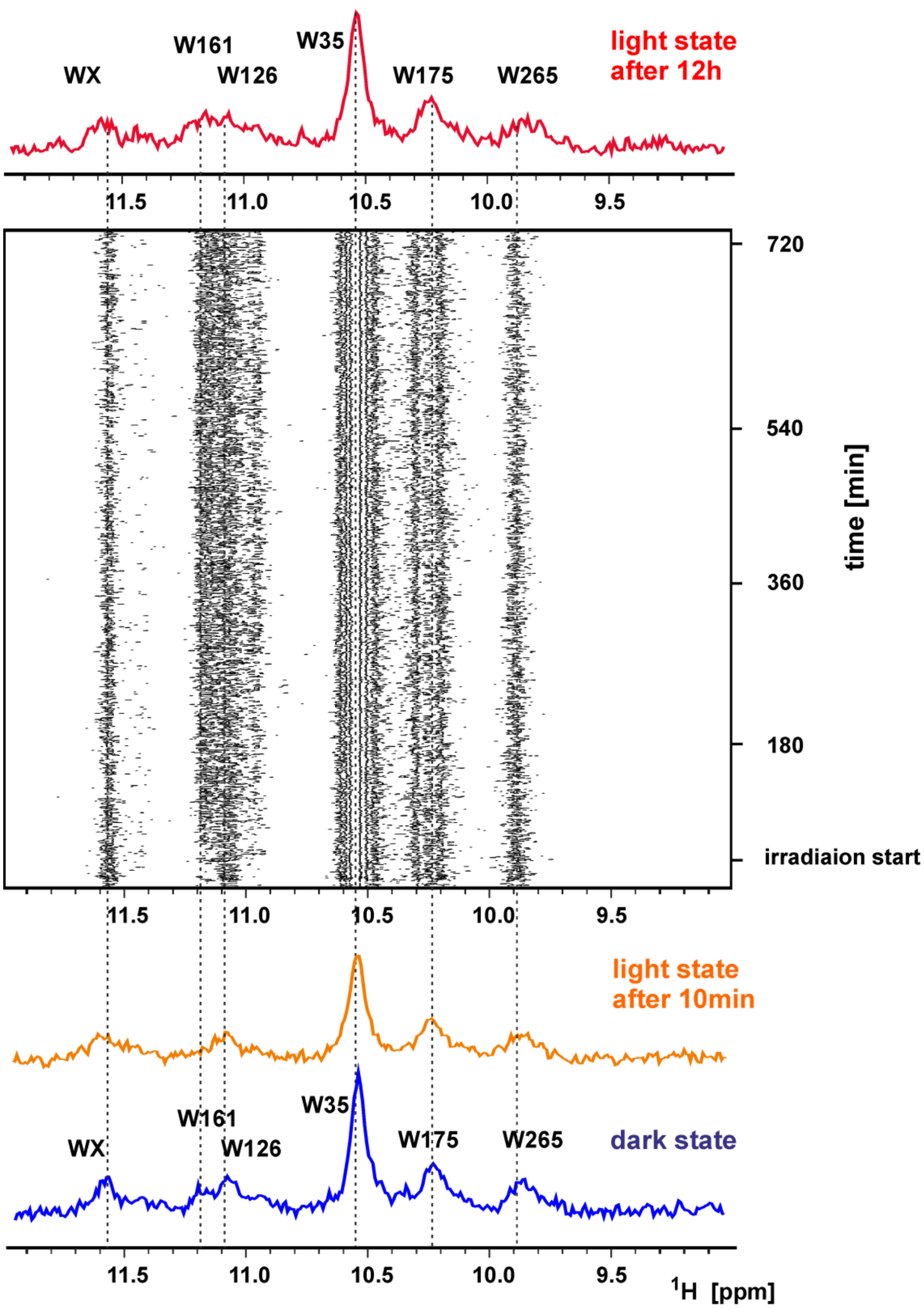

Figure S5. NMR kinetics of the G90D$_{S-S}$ mutant. A series of 1D ^{1}H NMR spectra of the G90D$_{S-S}$ mutant recorded at 800 MHz, 298 K, at different time intervals after illumination of 3 W for 0.5 seconds. The indole region of the spectrum is shown. The blue spectrum corresponds to the dark state recorded before the illumination, the orange one represents the light active state after 10 minutes and the red spectrum displays the indole resonances after 12 hours of experiment time. Due to the low signal-to-noise ratio, 16 1D planes recorded successively were summarized to represent red and orange spectra. No difference between red and orange spectra could be observed. The dash lines connect the chemical shift assignment of the indole tryptophan signals.

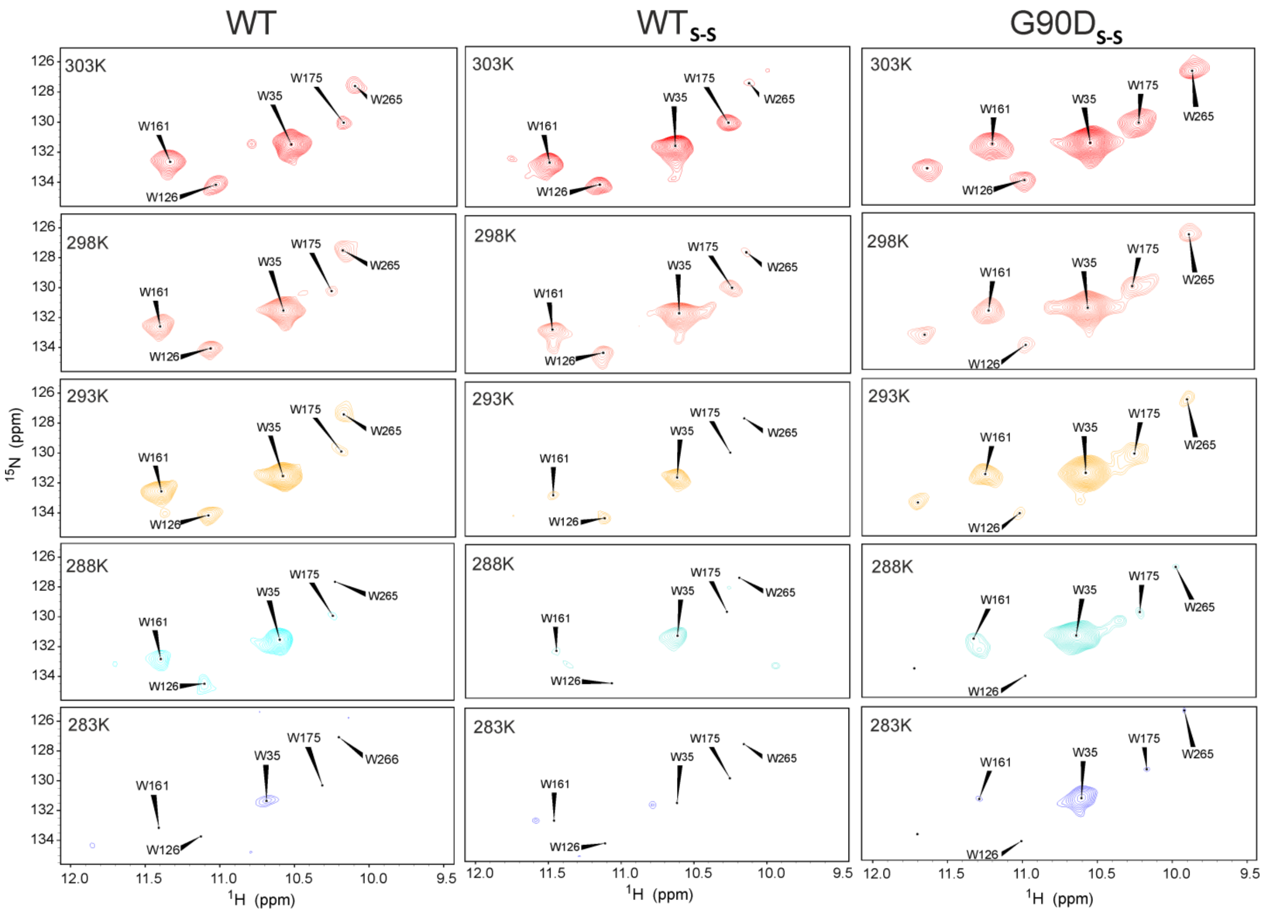

Figure S6. Temperature series of 2D SOFAST-HMQC NMR spectra of rhodopsin constructs in the dark state. All spectra were recorded at 600 MHz, 298 K under light dim conditions.

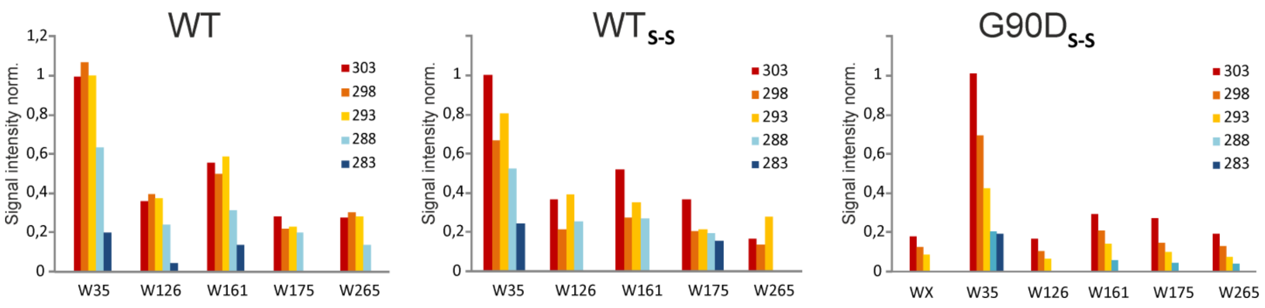

Figure S7. Graphical analysis of temperature series. Signal intensities from 2D SOFAST-HMQC NMR spectra of rhodopsin constructs recorded under light dim conditions were normalized to the highest W35 signal at 303 K.

2.3. Solid state MAS-NMR experiments

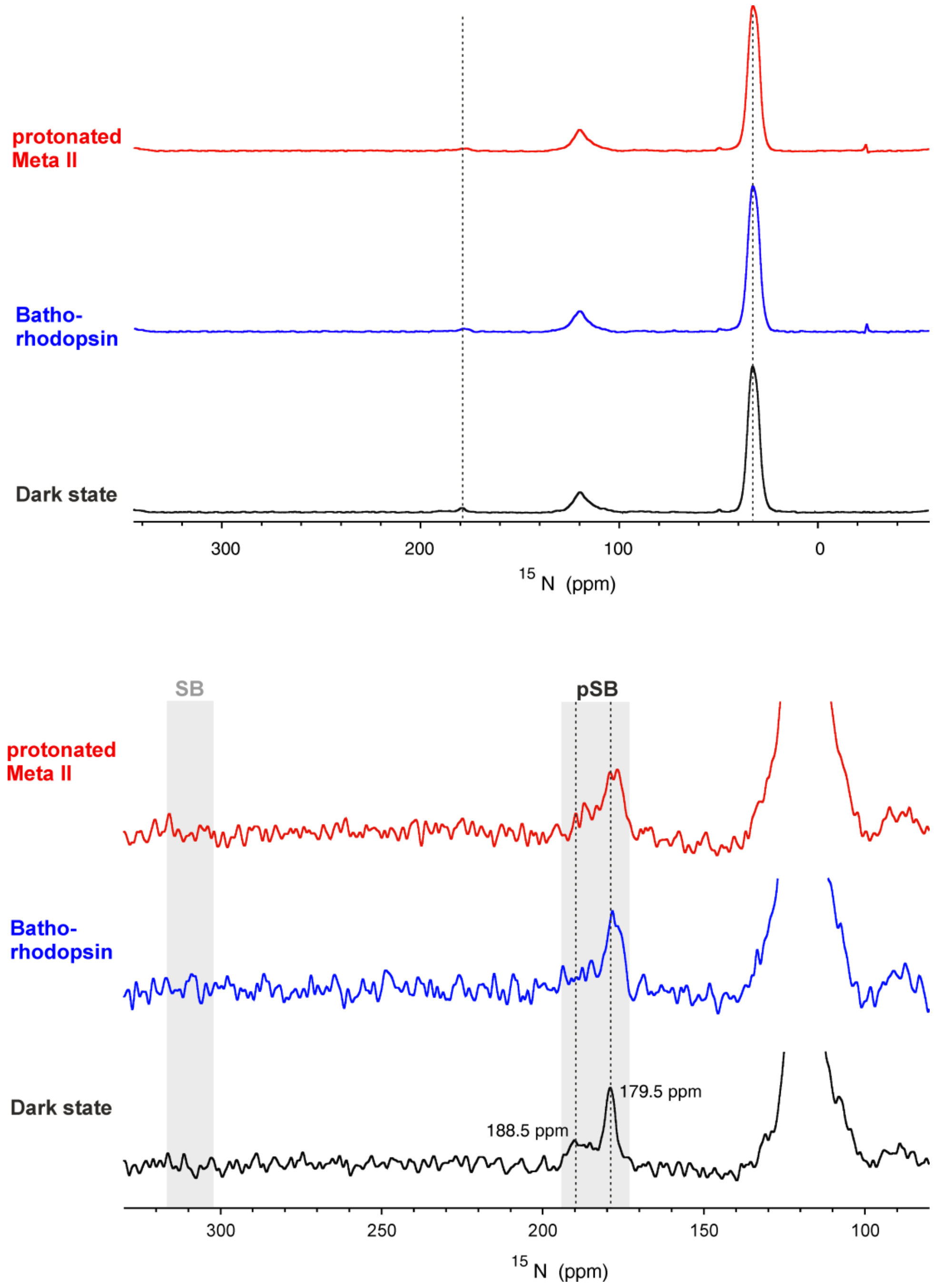

Figure S8. DNP-enhanced ^{15}N spectrum of ^{15}N lysine isotope labeled G90D$_{S-S}$ mutant in the dark state and under illumination. Three states were detected: dark state (black), bathorhodopin (blue) and light active Meta II (red) as discussed in the main text. The signal at 179.5 ppm, which corresponds to the protonated Schiff base, is visible in every state. The deprotonated Schiff base signal expected at 309 ppm is not detected in any state. On the bottom, spectra with full width are shown with the resonances of all lysine sidechains overlapping at around 38 ppm.

Table S1. Chemical shifts of the G90D$_{S\text{-}S}$ mutant observed for $^{13}C_2$ 14,15-retinal in the dark, bathorhodopsin and light active Meta II conformation. *Chemical shift perturbations are calculated as the difference between dark state and the corresponding intermediate conformation. Positive and negative sign reflects upfield and downfield shift of the light products, respectively.*

	C14, ppm	C15, ppm
Meta II state	132.6	168.1
Bathorhodpsin	119.8	169.6
Dark state	123.0	167.7

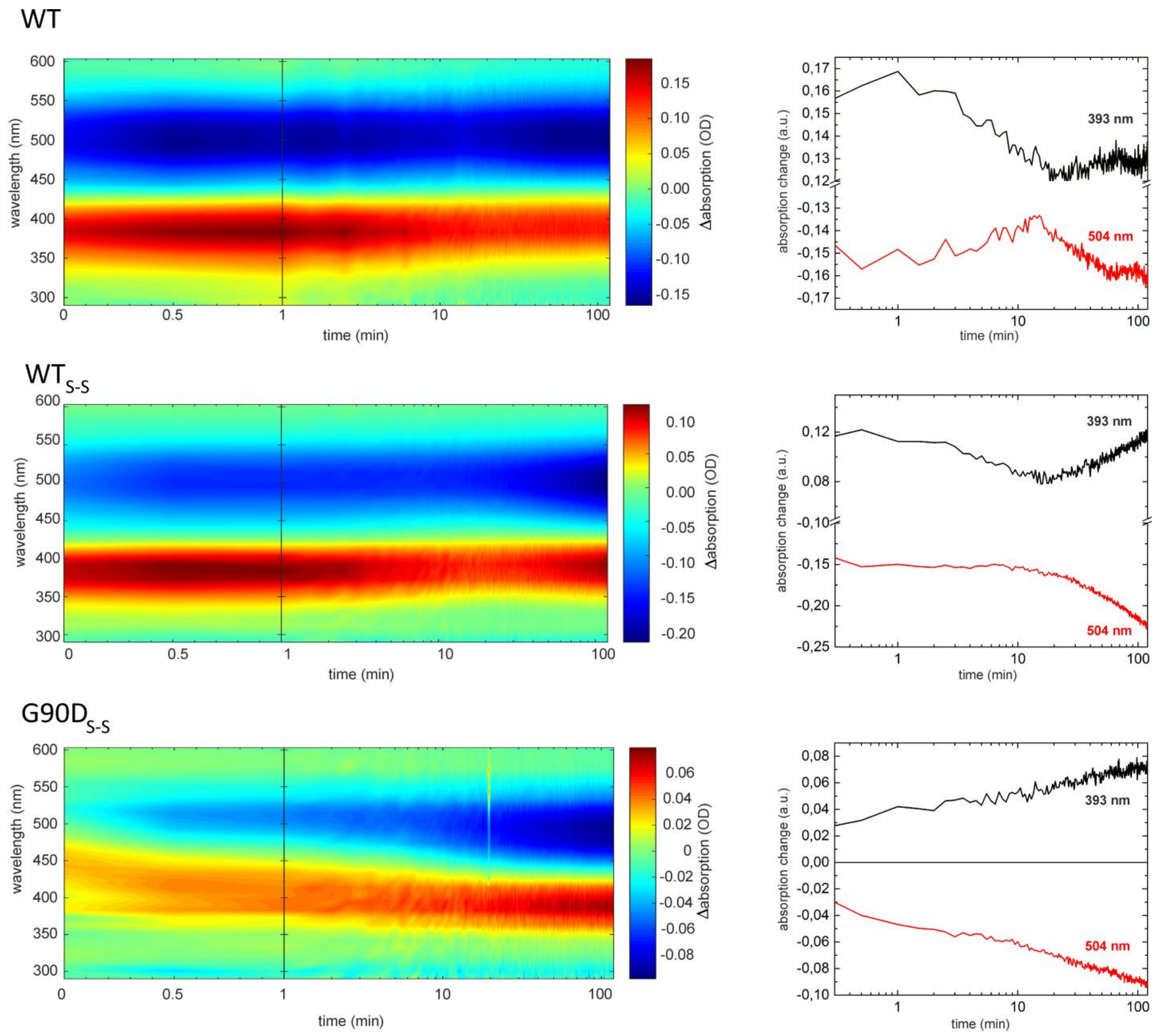

Figure S9. Flash photolysis experiments of WT (top), stabilized WT (middle) and the stabilized rhodopsin mutant G90D (bottom). The samples were solubilized in DDM (pH 6.5) and excited with a 490 nm (WT$_{S-S}$, G90D$_{S-S}$) or a 500 nm (WT) light pulse. It was measured in 0.5 min steps up to a delay time of 120 minutes. The transient absorbance changes (left) show two main bands, the negative ground state bleach (blue) and a spectrally blue shifted, positive band (red). Individual transients at characteristic wavelengths (right) show the drastically altered temporal behavior in G90D.

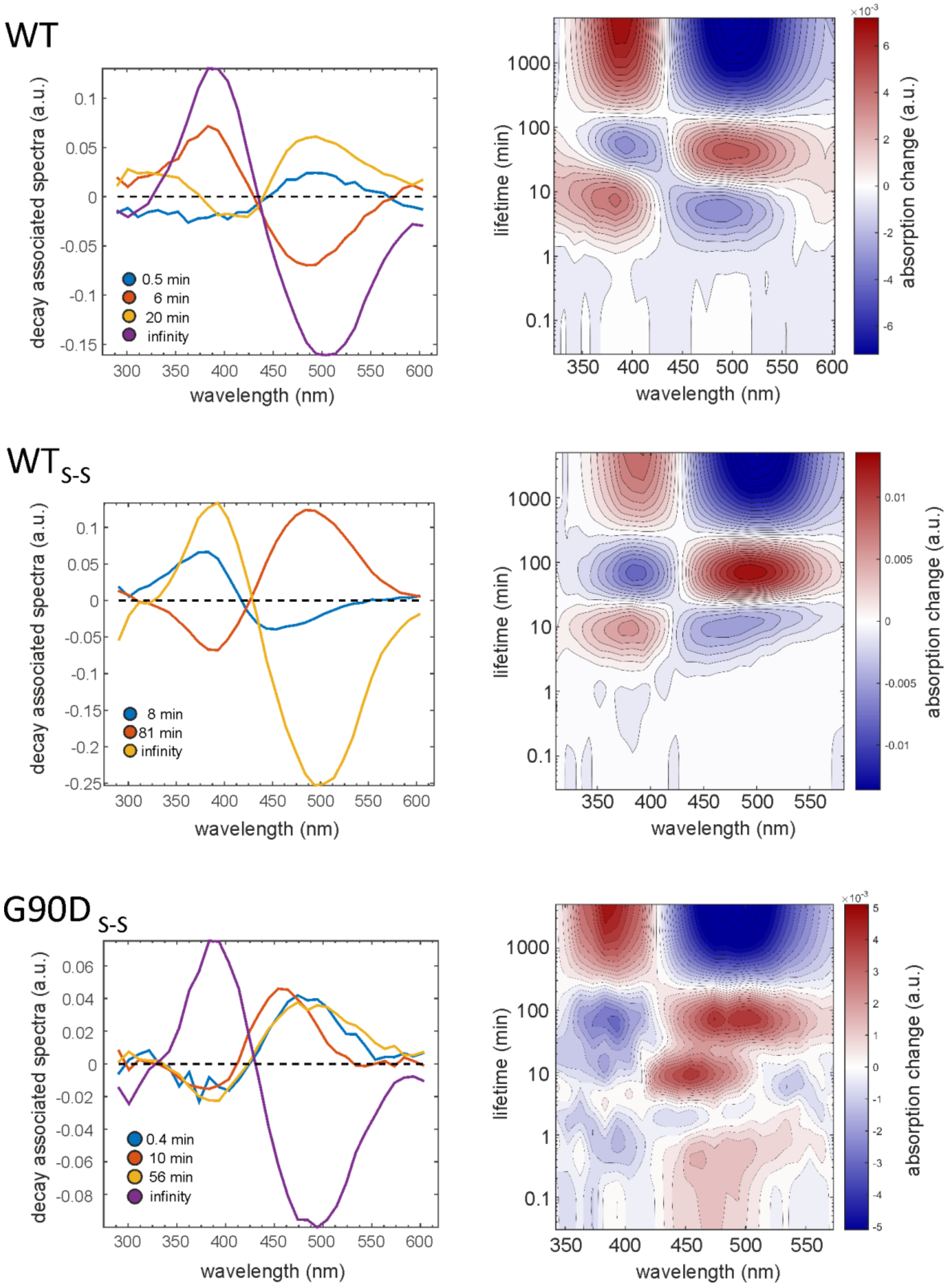

Figure S10. Decay associated spectra (left) and lifetime density maps (right) resulting from the global lifetime analysis of the transient absorption data (shown in Figure S9) of WT (top), stabilized WT (middle) and the stabilized rhodopsin mutant G90D (bottom). The kinetic analysis is the basis for the reaction model discussed in the main text.

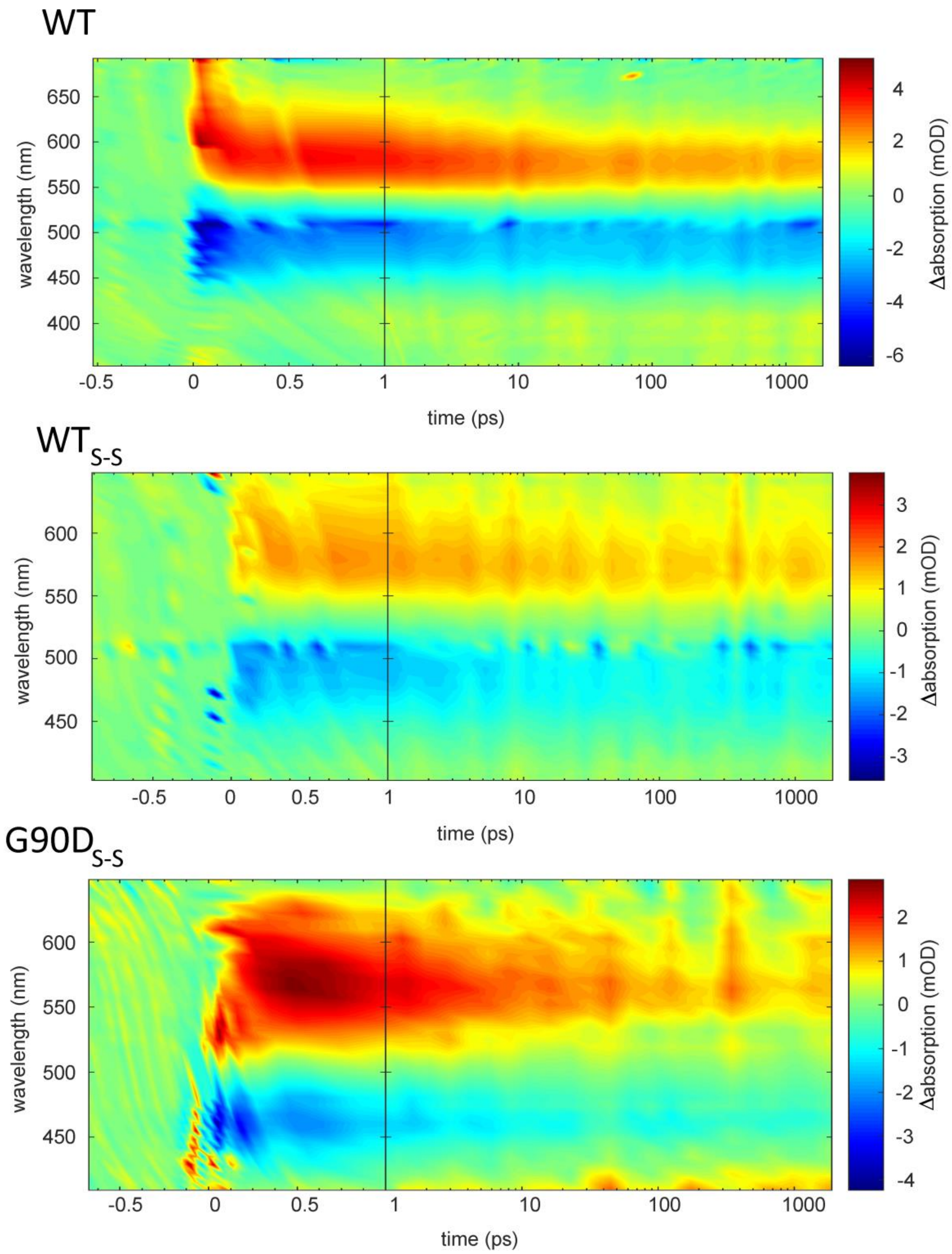

Figure S11. Early photodynamics of WT (top), stabilized WT (middle) and the stabilized rhodopsin mutant G90D (bottom). The samples were solubilized in DDM (pH 6.5) and excited with 50nJ pulses centered at 490 nm (G90D$_{S-S}$) or at 500 nm (WT, WT$_{S-S}$).

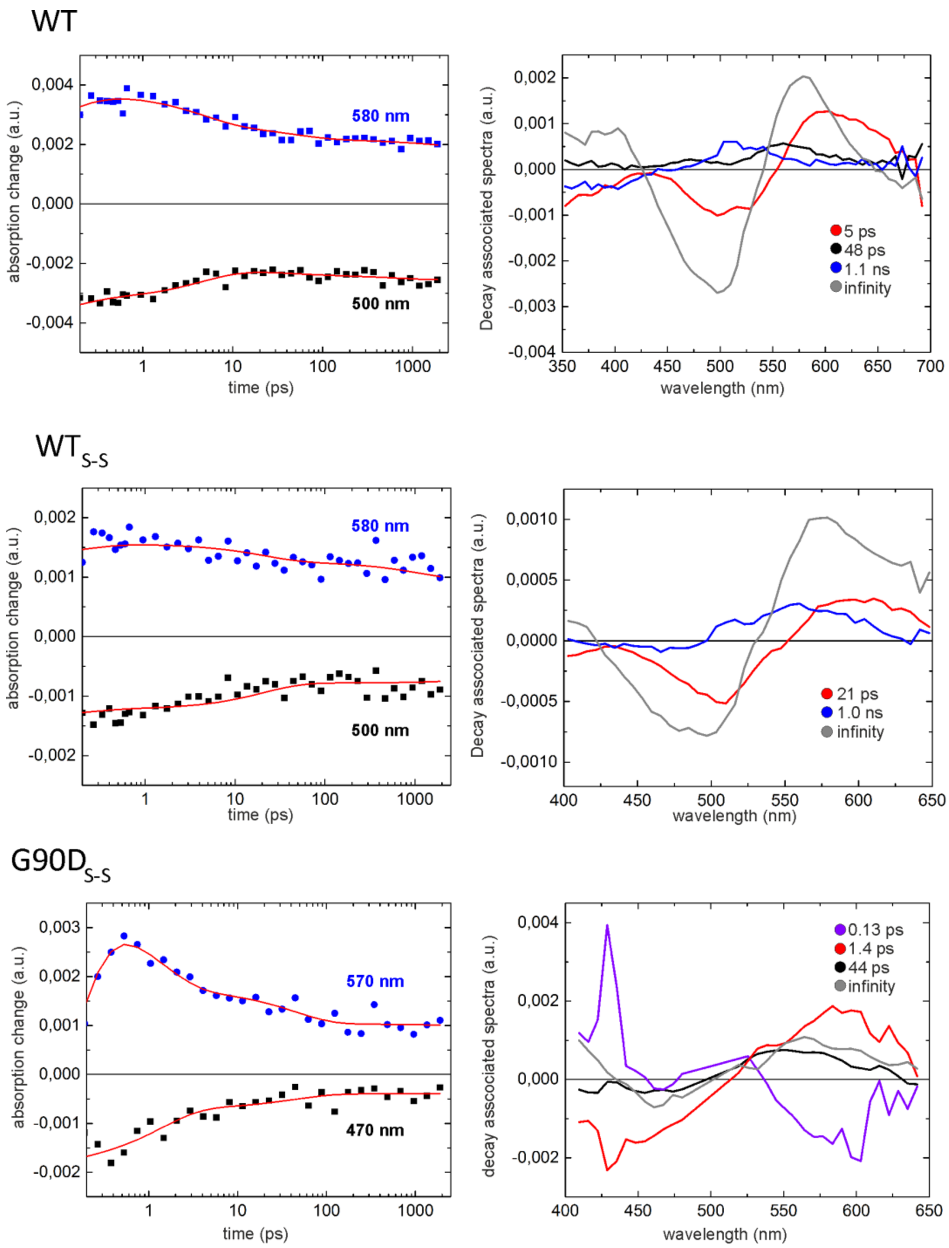

Figure S12. Individual transients at selected probing wavelengths (left) and decay associated spectra derived from the global lifetime analysis (right). The DAS spectrum of the 0.13ps component shows a negative structure around about 580 nm, which is assigned to the formation of photorhodopsin. This process could not be kinetically resolved in WT or $WT_{S\text{-}S}$. This shows that the formation of photorhodopsin is slowed down in the $G90D_{S\text{-}S}$. The kinetic analysis is the basis for the reaction model discussed in the main text.

3. <u>References</u>

[1] P. J. Reeves, J. M. Kim, H. G. Khorana, *Proc. Natl. Acad. Sci. U. S. A.* **2002**, *99*, 13413–13418.

[2] A. Singhal, M. K. Ostermaier, S. a Vishnivetskiy, V. Panneels, K. T. Homan, J. J. G. Tesmer, D. Veprintsev, X. Deupi, V. V Gurevich, G. F. X. Schertler, et al., *EMBO Rep.* **2013**, *14*, 520–6.

[3] P. J. Reeves, R. L. Thurmond, H. G. Khorana, *Proc. Natl. Acad. Sci. U. S. A.* **1996**, *93*, 11487–11492.

[4] A. Knowles, A. Priestley, *Vision Res.* **1978**, *18*, 115–116.

[5] J. Stehle, R. Silvers, K. Werner, D. Chatterjee, S. Gande, F. Scholz, A. Dutta, J. Wachtveitl, J. Klein-Seetharaman, H. Schwalbe, *Angew. Chemie - Int. Ed.* **2014**, *53*, 2078–2084.

[6] D. D. Oprian, R. S. Molday, R. J. Kaufman, H. G. Khorana, *Proc. Natl. Acad. Sci. U. S. A.* **1987**, *84*, 8874–8878.

[7] C. Slavov, N. Bellakbil, J. Wahl, K. Mayer, K. Rück-Braun, I. Burghardt, J. Wachtveitl, M. Braun, *Phys. Chem. Chem. Phys.* **2015**, *17*, 14045–14053.

[8] T. Wilhelm, J. Piel, E. Riedle, *Opt. Lett.* **1997**, *22*, 1494.

[9] E. Riedle, M. Beutter, S. Lochbrunner, J. Piel, S. Schenkl, S. Spörlein, W. Zinth, *Appl. Phys. B Lasers Opt.* **2000**, *71*, 457–465.

[10] C. Slavov, H. Hartmann, J. Wachtveitl, *Anal. Chem.* **2015**, *87*, 2328–2336.

[11] R. Croce, M. G. Müller, R. Bassi, A. R. Holzwarth, *Biophys. J.* **2001**, *80*, 901–915.

[12] K. Werner, I. Lehner, H. Kaur, C. Christian, C. Glaubitz, H. Schwalbe, J. Klein-Seetharaman, H. G. Khorana, *J. Biomol. NMR* **2007**, *37*, 303–312.

[13] M. Eilers, P. J. Reeves, W. Ying, H. G. Khorana, S. O. Smith, *Proc. Natl. Acad. Sci.* **1999**, *96*, 487–492.

[14] M. Hohwy, H. J. Jakobsen, M. Edén, M. H. Levitt, N. C. Nielsen, *J. Chem. Phys.* **1998**, *108*, 2686–2694.

[15] M. Hong, *J. Magn. Reson.* **1999**, *136*, 86–91.

[16] J. Becker-Baldus, C. Bamann, K. Saxena, H. Gustmann, L. J. Brown, R. C. D. Brown, C. Reiter, E. Bamberg, J. Wachtveitl, H. Schwalbe, *Proc. Natl. Acad. Sci.* **2015**, *112*, 9896–9901.

[17] M. Mehler, C. E. Eckert, A. J. Leeder, J. Kaur, T. Fischer, N. Kubatova, L. J. Brown, R. C. D. Brown, J. Becker-Baldus, J. Wachtveitl, et al., *J. Am. Chem. Soc.* **2017**, *139*, 16143-16153.
